# *Akkermansia muciniphila* Enhances Egg Quality and the Lipid Profile of Egg Yolk by Improving Lipid Metabolism

**DOI:** 10.3389/fmicb.2022.927245

**Published:** 2022-07-19

**Authors:** Fuxiao Wei, Xinyue Yang, Meihong Zhang, Chang Xu, Yongfei Hu, Dan Liu

**Affiliations:** State Key Laboratory of Animal Nutrition, College of Animal Science and Technology, China Agricultural University, Beijing, China

**Keywords:** laying hen, *Akkermansia muciniphila*, lipid metabolism, gut microbiota, egg yolk lipid

## Abstract

*Akkermansia muciniphila* (*A. muciniphila*) has shown potential as a probiotic for the prevention and treatment of non-alcoholic fatty liver disease in both humans and mice. However, relatively little is known about the effects of *A. muciniphila* on lipid metabolism, productivity, and product quality in laying hens. In this study, we explored whether *A. muciniphila* supplementation could improve lipid metabolism and egg quality in laying hens and sought to identify the underlying mechanism. In the first experiment, 80 Hy-Line Brown laying hens were divided into four groups, one of which was fed a normal diet (control group), while the other three groups were administered a high-energy, low-protein diet to induce fatty liver hemorrhagic syndrome (FLHS). Among the three FLHS groups, one was treated with phosphate-buffered saline, one with live *A. muciniphila*, and one with pasteurized *A. muciniphila*. In the second experiment, 140 Hy-Line Brown laying hens were divided into two groups and respectively fed a basal diet supplemented or not with *A. muciniphila* lyophilized powder. The results showed that, in laying hens with FLHS, treatment with either live or pasteurized *A. muciniphila* efficiently decreased body weight, abdominal fat deposition, and lipid content in both serum and the liver; downregulated the mRNA expression of lipid synthesis-related genes and upregulated that of lipid transport-related genes in the liver; promoted the growth of short-chain fatty acids (SCFAs)-producing microorganisms and increased the cecal SCFAs content; and improved the yolk lipid profile. Additionally, the supplementation of lyophilized powder of *A. muciniphila* to aged laying hens reduced abdominal fat deposition and total cholesterol (TC) levels in both serum and the liver, suppressed the mRNA expression of cholesterol synthesis-related genes in the liver, reduced TC content in the yolk, increased eggshell thickness, and reshaped the composition of the gut microbiota. Collectively, our findings demonstrated that *A. muciniphila* can modulate lipid metabolism, thereby, promoting laying hen health as well as egg quality and nutritive value. Live, pasteurized, and lyophilized *A. muciniphila* preparations all have the potential for use as additives for improving laying hen production.

## Introduction

Non-alcoholic fatty liver disease (NAFLD) encompasses a series of metabolic disorders characterized by hepatic lipid accumulation in the absence of excessive alcohol consumption. With a global prevalence of 22% to 29%, this disease accounts for a large proportion of chronic liver disease in adults worldwide (Cole et al., 2018). Fatty liver hemorrhagic syndrome (FLHS) is a metabolic disease similar to NAFLD that affects chickens, especially laying hens. FLHS is characterized by weight gain, excessive accumulation of hepatic and abdominal fat, severe hepatic steatosis, and inflammatory response, among other symptoms (Wu et al., 2019). Although the exact cause of this condition is unclear, it likely involves a combination of nutritional, genetic, environmental, and hormonal factors (Choi et al., 2012). In laying hens, FLHS impairs the ability of the liver to synthesize yolk precursors and follicle development, which, in turn, reduces the egg production rate as well as egg quality (Tumová et al., 2017). FLHS is also a leading cause of hen death in caged flocks, with one study reporting mortality rates ranging from 0.8% (young flocks) to 11.6% (old flocks) in some areas (Shini et al., 2019). Accordingly, to reduce mortality and maintain the productivity of laying hens, it is necessary to identify novel prevention and treatment strategies for FLHS.

Dysregulation of the “gut–liver axis” has been strongly implicated in the pathogenesis of both obesity and NAFLD (Arab et al., 2018). Gut dysbiosis and impairment of intestinal barrier function can result in bacterial products reaching the liver through the portal vein. Recognition of these products by specific receptors leads to the activation of pro-inflammatory responses, and, subsequently, the development of liver diseases, including NAFLD (Boursier and Diehl, 2016). Targeting the gut microbiota through the supplementation of probiotics and antibiotics, among other products, has met with some success in the treatment of NAFLD (Xue et al., 2017; Natividad et al., 2018), and thus represents a promising therapeutic strategy for patients with this disease.

The bacterium *Akkermansia muciniphila* (*A. muciniphila*) is an important constituent of the gut microbiota and has been proposed as a candidate next-generation probiotic, after *Lactobacillus* and *Bifidobacterium* (Zhang et al., 2019). Studies have shown that *A. muciniphila* abundance is significantly reduced in the cecal contents of NAFLD patients (Karlsson et al., 2012; Schneeberger et al., 2015; Shi et al., 2021), while a high abundance of *A. muciniphila* has been associated with better metabolic status, including improved blood glucose and lipid levels (Dao et al., 2016). *A. muciniphila* intervention can promote liver fat oxidation, inhibit liver lipid synthesis, reduce serum and liver lipid levels, relieve liver steatosis, and improve liver function (Everard et al., 2013; Zhao et al., 2017; Yang et al., 2020). It has also been reported to reduce body fat deposition and alleviate high-fat diet-induced obesity in mice (Ashrafian et al., 2019). There is evidence to support that *A. muciniphila* exerts its effects by stimulating mucus production; enhancing intestinal epithelial cell integrity and mucus layer thickness, thereby improving intestinal barrier function (Reunanen et al., 2015); inhibiting endotoxin production (Wu W. et al., 2017); and reducing chronic inflammation (Zhao et al., 2017). It is also known that mucin fermentation mediated by *A. muciniphila* results in the production of acetate and propionate in the intestinal mucus layer, resulting in both SCFAs being readily available to the host (Belzer and de Vos, 2012). Combined, these observations highlight that *A. muciniphila* intervention may represent an important means of treating NAFLD *via* its beneficial effects on obesity and lipid metabolism; however, research on this bacterium to date has mainly focused on humans and mice, and relatively little is known regarding the effects of *A. muciniphila* supplementation on lipid metabolism, productivity, and product quality in livestock and poultry.

The main purpose of this study was to evaluate the effect of *A. muciniphila* on lipid metabolism in laying hens with high-energy, low-protein diet-induced FLHS and in aged laying hens, as well as explore the underlying mechanisms. In addition, the effect of *A. muciniphila* on egg quality and lipid metabolite abundance in egg yolks was also investigated using untargeted lipidomics. The lipid profile of eggs can affect their added value and also has important implications for human health (Cui and Decker, 2016; Skórkowska-Telichowska et al., 2016; Yu et al., 2019a,b).

## Materials and Methods

### Preparation of Bacterial Suspensions and Lyophilized Powder

*A. muciniphila* strain (ATCC BAA-835) was cultured in modified Gifu Anaerobic Medium (GAM) broth (60 g/L). After incubation at 37°C under anaerobic conditions (10% H_2_, 10% CO_2_, 80% N_2_; Don Whitley Scientific DG250, West Yorkshire, United Kingdom) for 48 h, *A. muciniphila* cells were harvested by centrifugation at 8,000 rpm for 10 min at 4°C, resuspended in sterile pre-reduced phosphate-buffered saline (PBS, containing 20% glycerol), and finally stored at −80°C until use. Bacterial suspensions were diluted with sterile pre-reduced PBS to 3.5 ×10^8^ CFU/mL (final glycerol concentration: 2%) and activated in a water bath at 37°C for 10 min before use. Pasteurization consisted of heat treatment at 70°C for 30 min, as previously described (Plovier et al., 2017).

*A. muciniphila* fermentation broth, cultured as mentioned above, was centrifuged at 8,000 rpm for 10 min and the supernatant was discarded. A solution containing 10% skimmed milk powder was then added to the precipitate, followed by mixing and vacuum freeze-drying for 24 h. The obtained lyophilized powder was stored at −20°C. The *A. muciniphila* concentration in the fermentation broth before freeze-drying was 1 × 10^8^ CFU/ml.

### Animals and Experimental Design

#### Experiment 1: Induction of High-Energy, Low-Protein Diet-Induced FLHS in Laying Hens

A total of eighty Hy-Line Brown laying hens (28 weeks of age, 2.01±0.10 kg body weight) were used in this experiment. All the hens were subjected to an acclimatization period of 1 week during which they were fed a normal diet. The hens were then randomly divided into four groups, with 10 replicates per group and two hens per replicate. The control group continued receiving a normal diet (ND) while the other three groups were fed a high-energy, low-protein diet (a high-fat diet [HFD]) throughout the experiment to induce FLHS (Qiu et al., 2021). Among the three FLHS groups, one was administered 5 mL of sterile PBS by daily gavage (HFD group), one was treated with 5 ml of live *A. muciniphila* (HFD_LA) (3.5×10^8^ CFU/ml), and one received 5 ml of pasteurized *A. muciniphila* (HFD_PA) (3.5×10^8^ CFU/ml). The ND group was administered an equal volume of sterile PBS by daily gavage. The experiment lasted for 18 weeks (weeks 29–46). All the birds were raised in three-tier battery cages under a 16 h light/8 h dark photoperiod. The room temperature was maintained between 18 and 25°C. All the birds were allowed free access to water and mash feed. The ingredients and nutrient levels of the diets are presented in Table 1.

**Table 1:** Composition and nutritional levels of the normal and high-energy low-protein diet of FLHS laying hens.

**Items**	**Normal diet**	**High-energy low-protein diet**
**Ingredients**	**Content (%)**	
Corn	62.050	62.050
Soybean meal	23.430	14.910
Lime stone	8.470	8.470
Wheat bran	2.460	4.000
Calcicum hydrophosphate	1.250	1.340
Zeolite powder	0.560	0.724
Sodium chloride	0.350	0.350
Multimineral^a^	0.250	0.250
Multivitamin^b^	0.080	0.080
Ethoxyquin	0.030	0.030
DL-Methionine	0.090	0.146
L-Lysine hydrochloride 78%	/	0.320
Choline chloride 50%	0.080	0.080
Soybean oil	0.900	/
Lard	/	7.250
Total	100.000	100.000
**Nutrient levels**		
Metabolizable energy (mc/kg)	2.700	3.090
Crude protein (%)	16.501	13.000
Crude fat (%)	3.501	9.656
Calcium (%)	3.500	3.500
Available phosphorus (%)	0.333	0.333
Methionine (%)	0.347	0.347
Lysine (%)	0.859	0.859
Nitrogen-free extract (%)	52.894	51.256

a *The multimineral was composed of the following per kg diet: Mn, 60 mg; Fe, 80 mg; Cu, 8 mg; I, 1.2 mg; Se, 0.3 mg*.

b *The multivitamin was composed of the following per kg diet: vitamin A, 12,500 IU; vitamin D_3_, 32,500 IU; vitamin E, 18.75 mg; vitamin K_3_, 2.65 mg; vitamin B_1_, 2 mg; vitamin B_2_, 6 mg; vitamin B_12_, 0.025 mg; pantothenic acid, 12 mg; niacin, 50 mg; folic acid, 1.25 mg; biotin, 0.325 mg*.

At the end of week 18 of the experiment, blood was taken aseptically from the wing vein of one hen randomly selected from each cage. The blood was centrifuged at 3,000 rpm for 10 min at 4°C and the serum was collected and stored at −20°C. After serum collection, the hens were immediately euthanized *via* cutting the jugular vein. The liver was imaged and weighed and approximately 1 cm of liver tissue was taken and fixed in 4% paraformaldehyde for sectioning. A small piece of tissue was taken to determine the fat content of the liver; another tube of tissue samples was taken for mRNA analysis. The heart and abdominal fat were weighed. Collected cecal digesta samples were snap-frozen in liquid nitrogen and stored at −80°C for microbial analysis and detection of short-chain fatty acids (SCFAs) contents.

#### Experiment 2: Aged Laying Hens

For this experiment, 200 healthy Hy-Line Brown laying hens (64 weeks old) were used. After 8 h of starvation, blood was taken from the wing vein and centrifuged at 3,000 rpm for 10 min at 4°C for serum collection. The serum was used to assess total cholesterol (TC) and triglyceride (TG) contents. Based on the serum TC and TG contents and egg production rate, 140 chickens were selected and randomly divided into a control group and an *A. muciniphila* group, with seven replicates per group and 10 hens per replicate. The initial body weight (control group: 2.17 ± 0.12 kg; *A. muciniphila* group: 2.16 ± 0.12 kg), egg production rate (control group: 89.6 ± 5.67%; *A. muciniphila* group: 89.6 ± 5.48%), serum TC (control group: 3.71 ± 0.42 mmol/L; *A. muciniphila* group: 3.71 ± 0.40 mmol/L), and serum TG (control group: 8.59 ± 2.80 mmol/L; *A. muciniphila* group: 8.59 ± 2.74 mmol/L) were consistent between the two groups. All the hens were acclimated to the diet and environment for 1 week. Subsequently, the hens were fed a basal diet respectively supplemented or not with 1×10^7^ CFU of lyophilized powder of *A. muciniphila* per gram of feed (approximately 1×10^9^ CFU per chicken per day) throughout the trial period (weeks 65–76). The ingredients and nutrient levels of the diets are presented in Table 2.

**Table 2:** Composition and nutritional levels of basal diets of aged laying hens.

**Ingredients**	**Content (%)**
Corn	63.810
Soybean meal	20.000
Lime stone	8.300
Corn gluten meal	3.500
Calcicum hydrophosphate	1.500
Soybean oil	1.300
L-Lysine hydrochloride 78%	0.649
Sodium chloride	0.350
Multimineral^a^	0.250
DL-Methionine	0.185
Choline chloride 50%	0.100
Multivitamin^b^	0.020
Ethoxyquin MAX	0.020
Phytase 10000	0.016
Total	100.000
**Nutrient levels**	
Metabolizable energy (mc/kg)	2.777
Crude protein (%)	16.445
Calcium (%)	3.500
Available phosphorus (%)	0.365
Methionine (%)	0.441
Lysine (%)	1.216
Methionine (%)	0.700

a *The multimineral was composed of the following per kg diet: Mn, 60 mg; Fe, 80 mg; Cu, 8 mg; I, 1.2 mg; Se, 0.3 mg*.

b *The multivitamin was composed of the following per kg diet: vitamin A, 12,500 IU; vitamin D_3_, 32,500 IU; vitamin E, 18.75 mg; vitamin K_3_, 2.65 mg; vitamin B_1_, 2 mg; vitamin B_2_, 6 mg; vitamin B_12_, 0.025 mg; pantothenic acid, 12 mg; niacin, 50 mg; folic acid, 1.25 mg; biotin, 0.325 mg*.

At the end of the experiment, blood was collected from the wing vein of all the hens for the determination of serum TC and TG contents. One hen with blood lipid levels close to the average was selected from each replicate for sampling. All the hens were euthanized by exsanguination and the abdominal cavity was immediately opened. The liver and abdominal fat were photographed and weighed. The liver, cecal tissue, and cecal contents were snap-frozen in liquid nitrogen and stored at −80°C.

### Measurement of Serum Lipid Contents

Serum TC and TG contents were detected using an automatic biochemical analyzer (Kehua ZY KHB-1280, Beijing, China).

### Measurement of Hepatic Lipid Contents

Total lipids were extracted from pre-weighed liver samples using the chloroform–methanol–water method (Folch, 1957). The extracted lipids were dried in a centrifugal vacuum concentrator (Jiaimu CV200, Beijing, China) and then dissolved in PBS containing 5% Triton X-100. TC and TG contents were determined using a commercially available kit (Biosino, Beijing, China).

### Histological Evaluation

Paraformaldehyde-fixed liver tissues were embedded in paraffin, sectioned, stained with hematoxylin and eosin, and finally analyzed and imaged under a microscope (Leica DM750, Nussloch, Germany).

### Total RNA Isolation and Real-Time PCR

Total RNA was extracted from liver samples with Trizol Reagent (Beyotime, Shanghai, China) following the manufacturer's instructions. After the determination of concentration and quality, the extracted RNA was reverse transcribed into cDNA using the BeyoRT^TM^ II cDNA Synthesis Kit (with gDNA Eraser) (Beyotime). qPCR was performed with BeyoFast^TM^ SYBR Green qPCR Mix (2X, Low ROX) (Beyotime) on a QuantStudio^TM^ 7 Flex Real-Time PCR System (Applied Biosystems, Foster City, CA, United States). Relative mRNA abundance was calculated using the 2^−Δ*ΔCt*^ method with β-actin serving as the reference. The primers used for PCR (Sangon, Shanghai, China) are listed in Table 3.

**Table 3:** Primer sequence of target genes.

**Target genes**	**Forward primer (5^**′**^-3^**′**^)**	**Reserve primer (3^**′**^-5^**′**^)**
*β-actin*	GAGAAATTGTGCGTGACATCA	CCTGAACCTCTCATTGCCA
*FAS*	CCAACGATTACCCGTCTCAA	CAGGCTCTGTATGCTGTCCAA
*GPAT*	TCCATCGAGACCTAATGATAC	TAGACATCACAGCACAGGAC
*SCD1*	CCAGCGGAGATACTACAAGCC	CCGATTGCCAAACATGTGAGC
*SREBP1c*	CCCGAGGGAGACCATCTACA	GGTACTCCAACGCATCCGAA
*LXRα*	CAAAGGGAATGAATGAGC	AGCCGAAGGGCAAACAC
*HMGCR*	CATAGGTGGCTACAACG	TACGCTCCATCAAAGTG
*ABCA1*	TCAATCACCCGCTCAACT	CTGGCAGGAACAAAGGAC
*L-FABP*	GAAGAGTGTGAGATGGAGCTGCTG	GGTGATGGTGTCTCCGTTGAGTTC
*PPAR-α*	TGTGGAGATCGTCCTGGTCT	CGTCAGGATGGTTGGTTTGC
*PPAR-γ*	GCAGGAACAGAACAAAGAAG	TGCCAGGTCACTGTCATCTA
*CHREBP*	GATGAGCACCGCAAACCAGAGG	TCGGAGCCGCTTCTTGTAGTAGG
*CPT1*	TCGTCTTGCCATGACTGGTG	GCTGTGGTGTCTGACTCGTT
*ACOX1*	ATGTCACGTTCACCCCATCC	AGGTAGGAGACCATGCCAGT
*FXR*	AGTAGAAGCCATGTTCCTCCGTT	GCAGTGCATATTCCTCCTGTGTC
*GPR43*	AACGCCAACCTCAACAAGTC	TGGGAGAAGTCATCGTAGCA
*GPR41*	GAAGGTGGTTTGGGAGTGAA	CAGAGGATTTGAGGCTGGAG

*β-actin, beta-actin; FAS, fatty acid synthase; GPAT, glycerol-3-phosphate acyltransferase; SCD1, stearoyl-CoA desaturase 1; SREBP-1c, sterol regulatory element-binding protein 1c; LXRα, liver X receptor alpha; HMGCR, 3-hydroxy-3-methylglutaryl-CoA reductase; ABCA1, ATP-binding cassette transporter A1; L-FABP, liver fatty acid binding protein; PPAR-α, peroxisome proliferator-activated receptor alpha; PPAR-γ, peroxisome proliferator-activated receptor gamma; CHREBP, carbohydrate response element-binding protein; CPT1, carnitine palmitoyl transferase 1; ACOX1, acyl-CoA oxidase 1; FXR, farnesoid X receptor; GPR43, G protein-coupled receptor 43; GPR41, G protein-coupled receptor 41*.

### High-Throughput Sequencing of Gut Microbiota

Bacterial genomic DNA (gDNA) was extracted from the cecal digesta of laying hens using the PSP Spin Stool DNA Plus Kit (Invitek GmbH, Berlin, Germany) following the manufacturer's instructions. The quality of the extracted gDNA was assessed by gel electrophoresis. The V3–V4 region of the 16S rRNA gene was PCR-amplified using the 341F (5′-ACTCCTACGGGRSGCAGCAG-3′) and 806R (5′-GGACTACVVGGGTATCTAATC-3′) primer pair. Paired-end sequencing was performed on an Illumina Hiseq-PE250 sequencing platform at the Institute of Microbiology, Chinese Academy of Sciences (Beijing, China). The generated raw data were denoised, merged, and clustered using DADA2. Amplicon Sequence Variants (ASVs) were clustered with 100% similarity. Species annotation was performed for each ASV using the Naive Bayes classifier method based on the SILVA database. The phylum and genus composition of the microorganisms was visualized using the ggplot package in R. The alpha-diversity of the gut microbiota was calculated using the vegan package for R. Principal coordinates analysis (PCoA) and non-metric multidimensional scaling (NMDS) were used to explore differences in community structure. Permutational multivariate analysis of variance (PERMANOVA) was used to test the statistical significance of the two principal components obtained from the PCoA and NMDS models.

### Determination of Short Chain Fatty Acids

The cecal digesta samples were diluted with distilled water (4× the volume) and centrifuged at 12,000 × *g* for 15 min at 4°C. Then, 1 ml of the supernatant was mixed with 0.2 ml of 25% metaphosphoric acid solution, allowed to stand at 4°C for 30 min, and centrifuged at 12,000 × *g* for 10 min at 4°C (Calik and Ergün, 2015). The resulting supernatant was filtered through a 0.22-μm membrane filter and the acetic acid, propionic acid, isobutyric acid, butyric acid, isovaleric acid, valeric acid, and total SCFAs concentrations in the supernatant were determined using a gas chromatograph–mass spectrometer (Agilent 5975C, Santa Clara, CA, United States).

### Measurement of Egg Quality

For Experiment 1 (FLHS), one egg from each replicate was randomly selected for interior and exterior quality testing at the end of week 18. Interior and exterior quality were determined on 3 consecutive days [eggs were collected for 3 days (three eggs per replicate)]. Egg weight, eggshell breaking strength, albumen height, Haugh unit, and yolk color were determined using the Nabel DET-6000 egg analyzer (Kyoto, Japan). Eggshell thickness was a mean value of measurements at three locations on the egg surface (air cell, equator, and sharp end) obtained using an eggshell thickness gauge (Orka TI-PVX, Ramat Hasharon, Israel). A Vernier caliper was used to measure the longitudinal diameter and the maximum transverse diameter of the eggs, and the ratio of the two was taken as the egg shape index. Egg yolks were separated and weighed to calculate their specific gravity.

For Experiment 2 (aged laying hens), three eggs from each replicate were randomly selected for interior and exterior quality testing at the end of week 12. Interior and exterior quality were determined on 3 consecutive days [eggs were collected for 3 days (nine eggs per replicate)].

Total lipids were extracted from pre-weighed yolk samples using isopropyl alcohol (4 ml/100 mg) (Hammad et al., 1996). After centrifugation at 3,000 rpm for 10 min, the supernatant was collected and lipid content was determined using a commercially available kit (Biosino).

### Untargeted Lipidomics Analysis

The egg yolk samples were mixed and vacuum freeze-dried for 72 h. Then, 0.05 g of egg yolk powder was weighed in a 2-ml glass tube, mixed with 900 μl of ice-cold chloroform/methanol (1:2, v/v), vortexed for 15 s, and stored on ice for 1 h in the dark. After adding 300 μl of ice-cold chloroform and 250 μl of ice-cold water, the samples were vortexed for 15 s, centrifuged at 9,000 rpm for 2 min, and the lower layer was transferred to a new container. The residual part of the upper layer was extracted twice with 500 μl of ice-cold chloroform, vortexed for 15 s, and centrifuged at 9,000 rpm for 2 min, while the lower layer was combined and dried by vacuum centrifugation. The dried samples were reconstituted with 300 μl of isopropanol, centrifuged at 15,000 rpm for 10 min, passed through a 0.22-μm membrane, transferred to the injection vial, and stored at −80°C until detection.

Lipid separation was performed with a 6545 LC/Q–TOF system (Agilent, Santa Clara, CA, United States) equipped with an Agilent ZORBOX Eclipse Plus C18 column (959758-902; 100 mm × 2.1 mm, 1.8 μm) and an inline filter (5067-6189, Agilent 1290 Infinity II; 0.3 μm). The column temperature was maintained at 45°C, the injection volume was 1 μl, and the autosampler temperature was set to 20°C. Mobile phase A was prepared by mixing 10 mM ammonium formate with 5 μm Agilent deactivator additive in 5:3:2 water: acetonitrile: 2-propanol ratio. Mobile phase B was 10 mM ammonium formate in 1:9:90 water: acetonitrile: 2-propanol. The flow rate was 0.4 ml/min.

Agilent Lipid Annotator software was used to create a search database. Then, Agilent Profinder software was used for processing the original mass spectrometry data, including retention time correction, peak identification, peak extraction, peak integration, and peak alignment, followed by CEF file output. Finally, statistical processing was undertaken using Agilent Mass Profiler Professional software.

### Statistical Analysis

All data were analyzed using SPSS version 22.0 (SPSS Inc., Chicago, IL, United States). Differences between two groups were analyzed using an independent samples *t*-test. For more than groups, one-way ANOVA was used, followed by Duncan's test for multiple comparisons. A *p*-value of <0.05 was considered significant and 0.05 ≤ *p* <0.10 was considered a tendency.

## Results

### *A. muciniphila* Attenuated FLHS in Laying Hens

**Figure 1:**
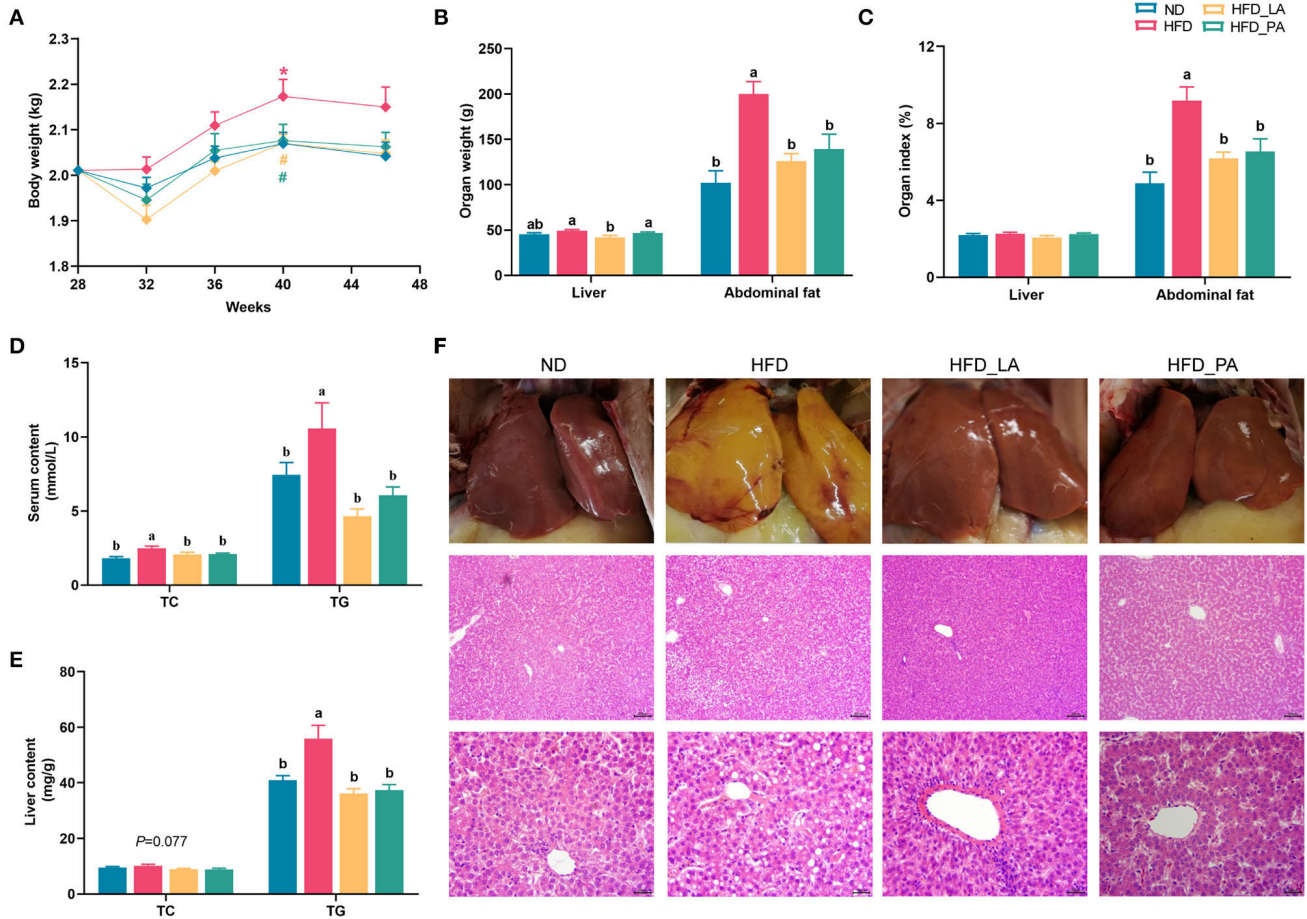
*Akkermansia muciniphila* attenuated fatty liver hemorrhagic syndrome (FLHS) induced by a high-energy, low-protein diet (high-fat diet, HFD) in laying hens. **(A)** The body weight of laying hens (*n* = 20). *p < 0.05, compared with the normal diet (ND) group. # *p* < 0.05, compared with the HFD group. **(B)** Liver and abdominal fat weight (*n* = 10). **(C)** Liver and abdominal fat index (*n*=10). **(D)** Serum total cholesterol (TC) and triglyceride (TG) levels (*n* = 10). **(E)** Hepatic TC and TG levels (*n* = 10). **(F)** Examination of liver pathology using hematoxylin and eosin (H&E) staining. Different letters indicate significant differences at *p* < 0.05.

Compared with the ND group, the laying hens in the HFD group showed a significant increase in body weight at week 40 and abdominal fat deposition, serum TC and TG levels, and hepatic TG concentrations at the end of the experiment (all *p* < 0.05) (Figures 1A–E). Notably, in laying hens with HFD-induced FLHS, treatment with either live or pasteurized *A. muciniphila* decreased body weight and abdominal fat mass gain, and also attenuated hepatic steatosis, as evidenced by decreased serum TC and TG contents and liver TG content (*p* < 0.05) (Figures 1B–E). Unexpectedly, liver weight was markedly decreased in the HFD_LA group when compared with that in the HFD group (*p* < 0.05) (Figure 1B). Assessment of liver pathology using H&E staining further confirmed the ameliorative effect of *A. muciniphila* on FLHS-related phenotypes in laying hens (Figure 1F). Compared with the ND group, the livers of hens in the HFD group were enlarged and lighter; they were also yellowish and exhibited disordered hepatic cell arrangement, hepatocyte karyolysis, and a great number of fat vacuoles, suggestive of severe FLHS. In contrast, the livers of laying hens treated with live or pasteurized *A. muciniphila* displayed a smooth, red–brown external surface, and fewer and smaller vacuoles, which was similar to that observed in the ND group.

### *A. muciniphila* Decreased Lipid Synthesis and Increased Lipid Transport in the Liver of Laying Hens With HFD-Induced FLHS

**Figure 2:**
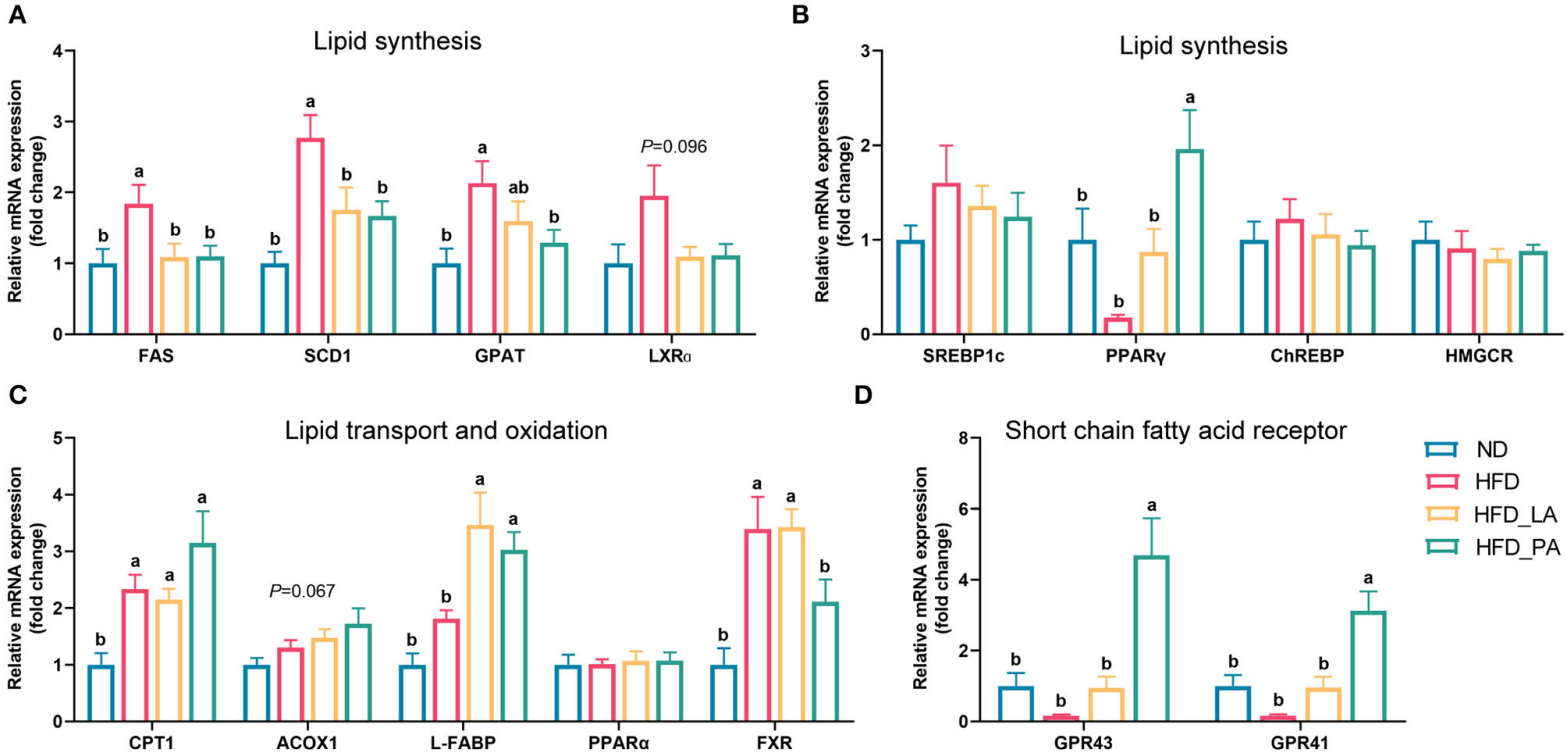
Effects of *Akkermansia muciniphila* treatment on the relative mRNA expression of lipid metabolism-related genes in the livers of laying hens with fatty liver hemorrhagic syndrome (FLHS) (*n* = 10). **(A,B)** mRNA expression levels of genes involved in the regulation of lipid synthesis. **(C)** mRNA expression levels of genes involved in the regulation of lipid transport and oxidation. **(D)** Relative mRNA expression of SCFAs receptors. The mRNA expression levels in the normal diet (ND) group (controls) were set as 1, and the relative fold increases were determined by comparison with the ND group. Different letters indicate significant differences at *p* < 0.05. *FAS*, fatty acid synthase; *SCD1*, stearoyl-CoA desaturase 1; *GPAT*, glycerol-3-phosphate acyltransferase; *LXR*α, liver X receptor alpha; *SREBP-1c*, sterol regulatory element-binding protein 1c; *PPAR-*γ, peroxisome proliferator-activated receptor gamma; *CHREBP*, carbohydrate response element-binding protein; *HMGCR*, 3-hydroxy-3-methylglutaryl-CoA reductase; *CPT1*, carnitine palmitoyl transferase 1; *ACOX1*, acyl-CoA oxidase 1; *L-FABP*, liver fatty acid binding protein; *PPAR-*α, peroxisome proliferator-activated receptor alpha; *FXR*, farnesoid X receptor; *GPR43*, G protein-coupled receptor 43; *GPR41*, G protein-coupled receptor 41.

qPCR analysis of the liver of laying hens revealed that, compared with the ND, the high-energy, low-protein diet significantly increased the expression levels of genes related to lipid synthesis, such as fatty acid synthase (*FAS*), stearoyl-CoA desaturase 1 (*SCD1*), and glycerol-3-phosphate acyltransferase (*GPAT*) (all *p* < 0.05) (Figure 2A), and tended to increase the expression levels of liver X receptor-alpha (*LXRa*) (*p* = 0.096) (Figure 2B). The consumption of an HFD also markedly upregulated the expression levels of genes related to lipid transport and oxidation, such as carnitine palmitoyl transferase 1 (*CPT1*) and farnesoid X receptor (*FXR*) (both *p* < 0.05) (Figure 2C). Compared with those in the HFD group, laying hens in the HFD_LA group displayed markedly reduced expression levels of *FAS* and *SCD1* (both *p* < 0.05) (Figure 2A) and significantly upregulated expression levels of liver fatty acid-binding protein (*L*-*FABP*) (*p* < 0.05) (Figure 2C). Additionally, the expression levels of *LXR*α tended to decrease in the HFD_LA group (*p* = 0.096) (Figure 2A). Treatment with pasteurized *A. muciniphila* (HFD_PA group) significantly decreased the expression levels of *FAS, SCD1*, and *GPAT* (all *p* < 0.05) (Figure 2A); markedly increased the expression levels of peroxisome proliferator-activated receptor-gamma (*PPAR-*γ), *L*-*FABP*, and the SCFAs receptors *GPR43* and *GPR41* (all *p* < 0.05) (Figures 2B–D); and tended to downregulate the expression levels of *LXR*α (*p* = 0.096) (Figure 2A). Acyl-CoA oxidase 1 (*ACOX1*) mRNA expression tended to be upregulated in the *A. muciniphila* treatment groups relative to the ND group (*p* = 0.067) (Figure 2C).

### *A. muciniphila* Improved Egg Quality and the Yolk Lipid Profile in Laying Hens With HFD-Induced FLHS

**Figure 3:**
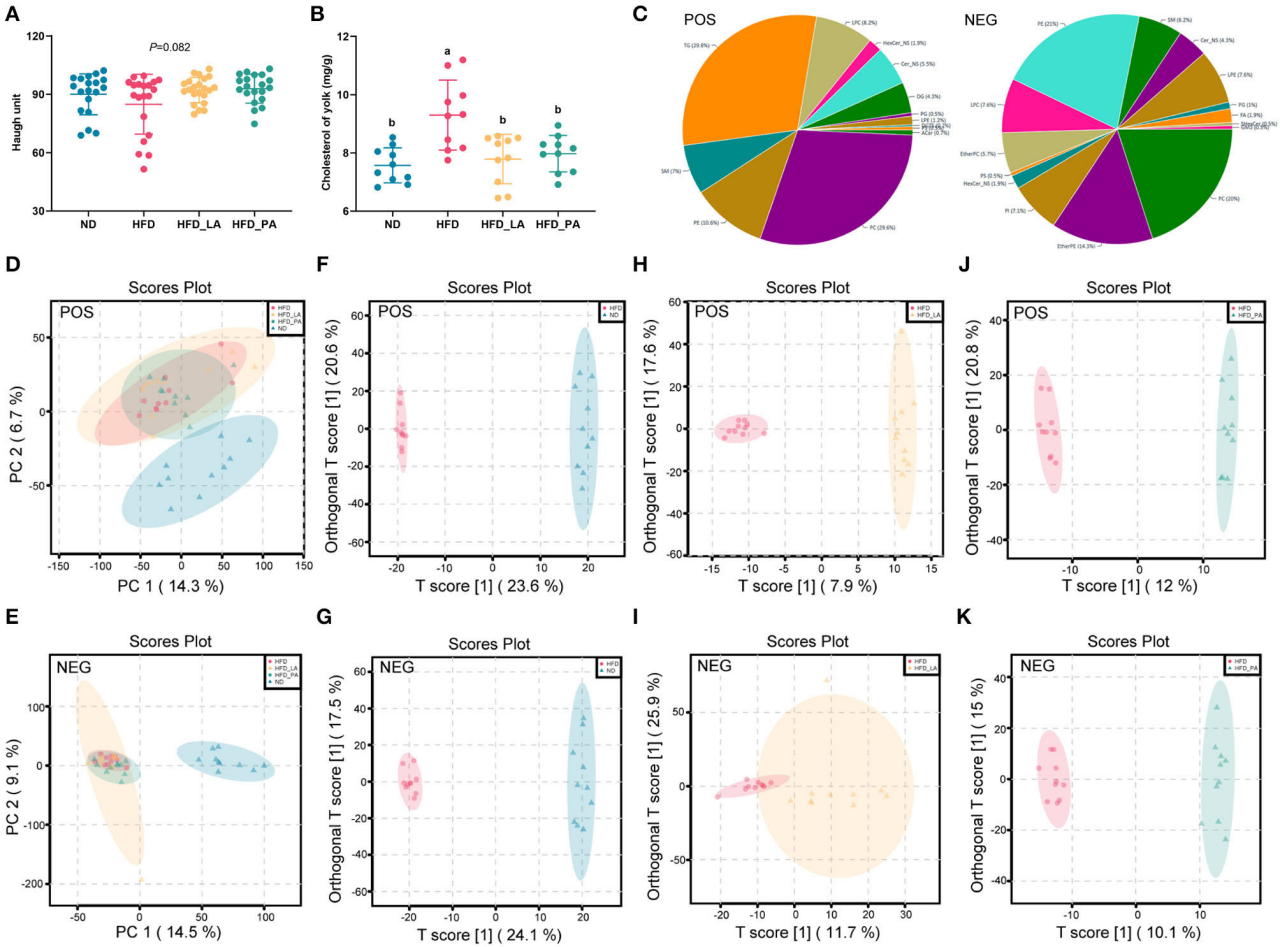
*Akkermansia muciniphila* improved egg quality and the lipid profile of egg yolks in laying hens with fatty liver hemorrhagic syndrome (FLHS). **(A)** Haugh unit (*n*=30). **(B)** Cholesterol content in the yolk (mg/g) (*n*=10). **(C)** Distribution of lipid classes and species identified in egg yolk samples from the four treatment groups. **(D,E)** Principal component analysis (PCA) score plots among the four treatment groups in positive ion (ES+) and negative ion (ES–) modes. **(F,G)** The orthogonal partial least squares discriminant analysis (OPLS-DA) score plots between the normal diet (ND) and high-fat diet (HFD) groups in ES+ and ES– modes. **(H,I)** The OPLS-DA score plots between the HFD and HFD + live *A. muciniphila* (HFD_LA) treatment groups in ES+ and ES– modes. **(J,K)** The OPLS-DA score plots between the HFD and HFD + pasteurized *A. muciniphila* (HFD_PA) treatment groups in ES+ and ES– modes. Different letters indicate significant differences at *p* < 0.05. **(C–K)** POS represents ES+ mode and NEG represents ES– mode (*n* = 1 0).

The high-energy, low-protein diet significantly increased the TC content of yolks as well as the egg shape index (both *p* < 0.05) (Figure 3B and Supplementary Table S1). Treatment with either live or pasteurized *A. muciniphila* markedly decreased the TC content of yolks (*p* < 0.05) (Figure 3B) and tended to increase the Haugh value (*p* = 0.082) (Figure 3A). Compared with the ND group, the HFD groups all had increased yolk color (*p* < 0.05) (Supplementary Table S1).

Untargeted lipidomics analysis identified a total of 3,374 lipid species in egg yolk samples of the four treatment groups. Of these, 1,648 (including 29.8% TGs, 29.6% phosphatidylcholines [PCs], 10.6% phosphatidylethanolamines [PEs], and 8.2% lyso-phosphatidylcholines [LPCs]) detected in positive ion mode (ES+) and 1,726 (including 21% PEs, 20% PCs, 7.6% LPCs, 7.6% lyso- phosphatidylethanolamines [LPEs]) detected in negative ion mode (ES–) (Figure 3C). To fully evaluate the changes occurring in the lipid profiles among the groups, each lipid in all the groups was further analyzed using principal component analysis (PCA), orthogonal partial least squares discriminant analysis (OPLS-DA), and volcano plot analysis, all performed in MetaboAnalyst 5.0.

PCA was used to investigate the differences in lipid profile between groups as well as the effect of *A. muciniphila* intervention on the egg yolk lipid profile. A striking separation was noted between the ND and HFD groups in both the ES+ and ES– patterns (Figures 3D,E and Supplementary Figures S1A,B). Furthermore, neither the HFD_LA group nor the HFD_PA group formed a clear cluster with the HFD group (Supplementary Figures S1C–F).

To determine which lipids showed differential abundance among the groups, OPLS-DA was used to define the differentially abundant metabolites between the HFD group and the ND, HFD_LA, and HFD_PA groups. The score plots in ES+ and ES– indicated that there were significant differences between the HFD group and each of the other three groups (Figures 3F–K). The lipids displaying significant differences in abundance (potential lipid markers) were further screened according to volcano plots (fold change >2 and <0.5, *p* < 0.05, and VIP [variable importance in projection] score >1) (Supplementary Figure S2). Based on these criteria, 366 annotated lipid markers were identified as showing differential abundance between the HFD and ND groups, with PCs and PEs accounting for the largest proportion. Twenty-six types of PC and 16 types of PE showed higher abundance, while 55 types of PC and 31 types of PE exhibited lower abundance in the HFD group compared with the ND group (Supplementary Table S2). The lipids displaying differential abundance between the HFD and HFD_LA groups comprised 10 TG metabolites, which were all downregulated in the HFD_LA group, and 14 PE metabolites, 8 of which were upregulated in the HFD_LA group (Supplementary Table S3); 54 annotated TG metabolites were identified between the HFD and HFD_PA groups, and 15 types were upregulated and 39 types were downregulated (Supplementary Table S4).

### *A. muciniphila* Supplementation Changed the Composition of the Gut Microbiota in Laying Hens With HFD-Induced FLHS

**Figure 4:**
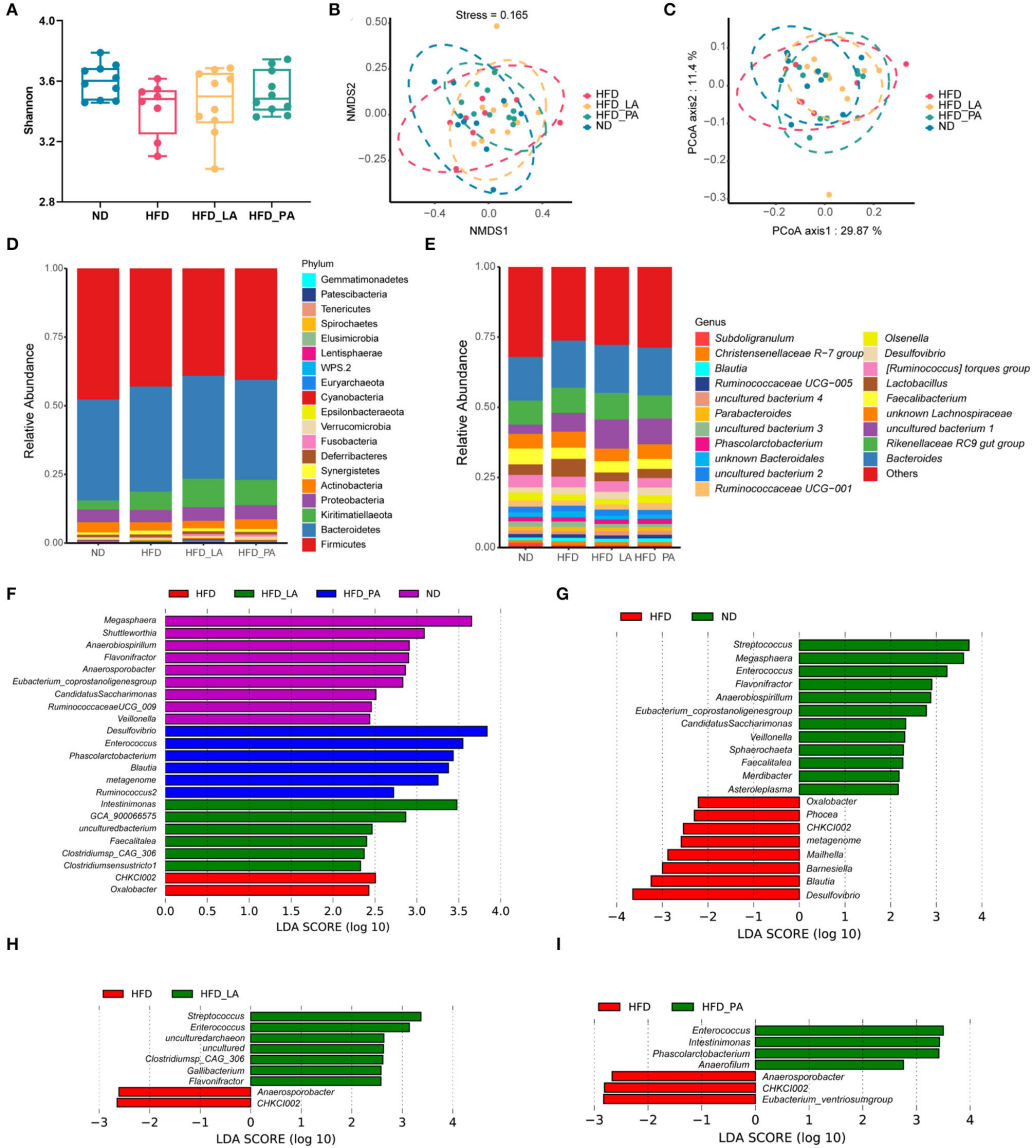
*Akkermansia muciniphila* changed the composition of the gut microbiota in laying hens with fatty liver hemorrhagic syndrome (FLHS) (*n* = 10). **(A)** Determination of gut microbiota diversity (as measured by the Shannon index). **(B)** Non-metric multidimensional scaling (NMDS) analysis. **(C)** Principal coordinates analysis (PCoA). **(D)** Taxonomic analysis of the gut microbiota at the phylum level. **(E)** Taxonomic analysis of the gut microbiota at the genus level. **(F)** The linear discriminant analysis (LDA) score shows a significant difference in bacterial composition among the four treatment groups. **(G)** The LDA score shows a significant difference in bacterial composition between high-fat diet (HFD)- and normal diet (ND)-fed laying hens. **(H)** The LDA score shows a significant difference in bacterial composition between HFD- and HFD + live *A. muciniphila* (HFD_LA)-fed laying hens. **(I)** The LDA score shows a significant difference in bacterial composition between HFD- and HFD + pasteurized *A. muciniphila* (HFD_PA)-fed laying hens. Different letters indicate significant differences at *p* < 0.05.

No significant changes in alpha-diversity were observed (as measured by the Shannon index) (Figure 4A) and no significant separations of cecum microbial communities were detected (as determined by both PCoA and NMDS plot analysis) (Figures 4B,C) among the four groups. Linear discriminant analysis (LDA) effect size (LEfSe) results revealed that several beneficial bacteria, such as members of the genera *Streptococcus, Enterococcus, Clostridium* sp. CAG:306, *Gallibacterium*, and *Flavonifractor*, were significantly enriched (LDA score >2, *p* < 0.05) in the HFD_LA group relative to the HFD group (Figure 4H). Additionally, members of the genera *Enterococcus, Phascolarctobacterium, Intestinimonas*, and *Anaerofilum* were enriched in the HFD_PA group when compared with the HFD group (Figure 4I). Intriguingly, the genus *Enterococcus* became the common predominant microbe among the ND, HFD_LA, and HFD_PA groups (Figures 4G–I).

### *A. muciniphila* Increased the Cecal SCFAs Content in Laying Hens With HFD-Induced FLHS

**Figure 5:**
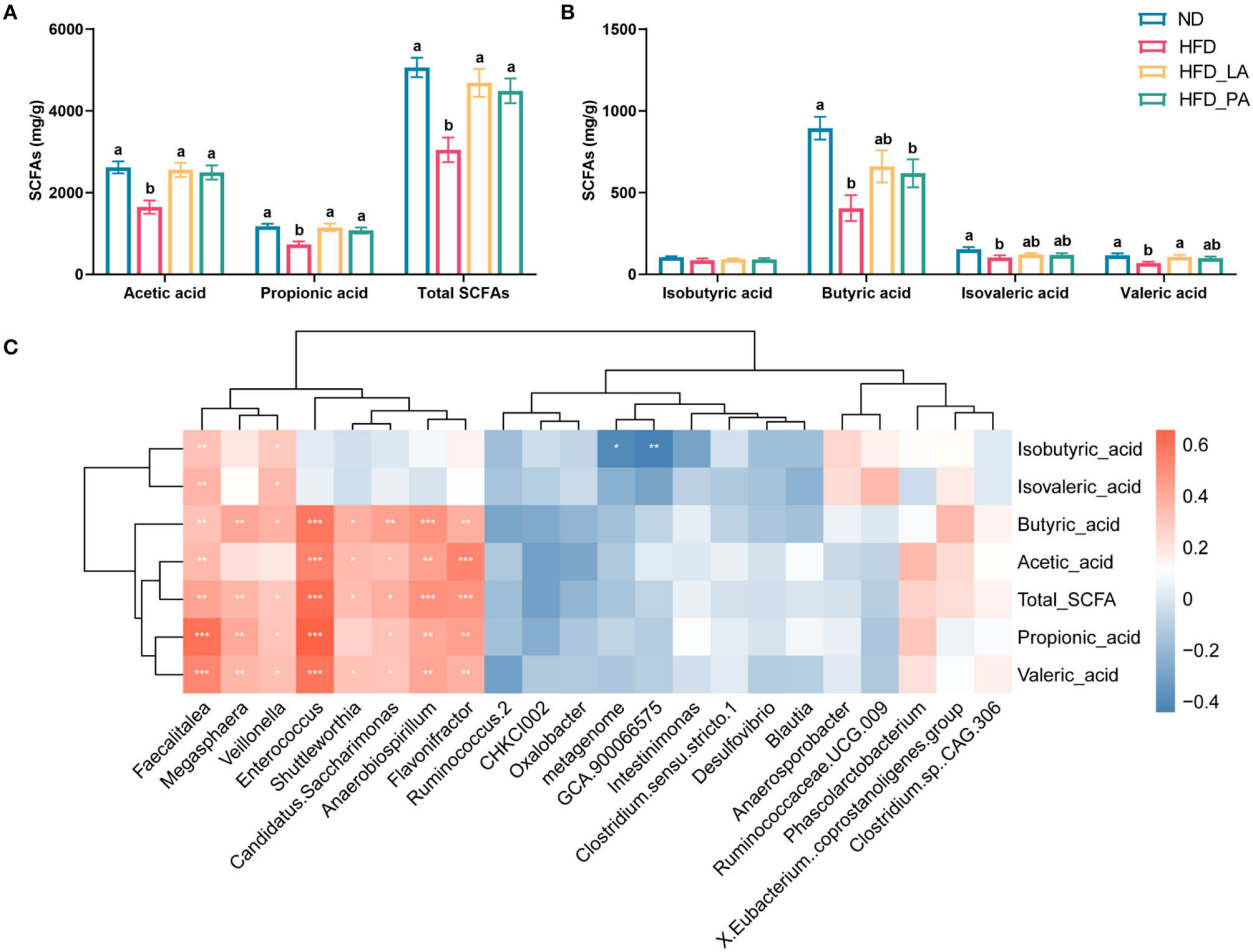
*Akkermansia muciniphila* treatment increased the cecal short-chain fatty acid (SCFA) content in laying hens with fatty liver hemorrhagic syndrome (FLHS) (*n* = 10). **(A,B)** SCFA contents in the cecum. Different letters indicate significant differences at *p* < 0.05. **(C)** Correlation analysis between SCFA contents and the microbial species showed significant differential abundance between groups. Red represents positive correlations and blue represents negative correlations. *p < 0.05, **p < 0.01, ***p < 0.001.

Compared with the ND group, the HFD group had significantly lower cecal concentrations of SCFAs, including acetic acid, propionic acid, butyric acid, isovaleric acid, valeric acid, and total SCFAs; however, treatment with either live or pasteurized *A. muciniphila* markedly attenuated the reduction in the levels of acetic acid, propionic acid, and total SCFAs seen in the HFD group (*p* < 0.05) (Figures 5A,B). Live *A. muciniphila* also promoted valeric acid production, while pasteurized *A. muciniphila* enhanced the production of butyric acid (*p* < 0.05) (Figure 5B). Correlation analysis of SCFAs contents and the microbial species displaying significant differential abundance between groups further confirmed the ability of *A. muciniphila* to promote the growth of acid-producing microbes, such as *Faecalitalea, Flavonifractor*, and *Enterococcus*. Several other bacteria that were positively correlated with the SCFAs contents, such as *Megasphaera, Veillonella, Shuttleworthia, Candidatus, Saccharimonas*, and *Anaerobiospirillum*, were enriched in the ND group (Figure 5C).

### *A. muciniphila* Lyophilized Powder Supplementation Ameliorated Lipid Metabolism in Aged Laying Hens

**Figure 6:**
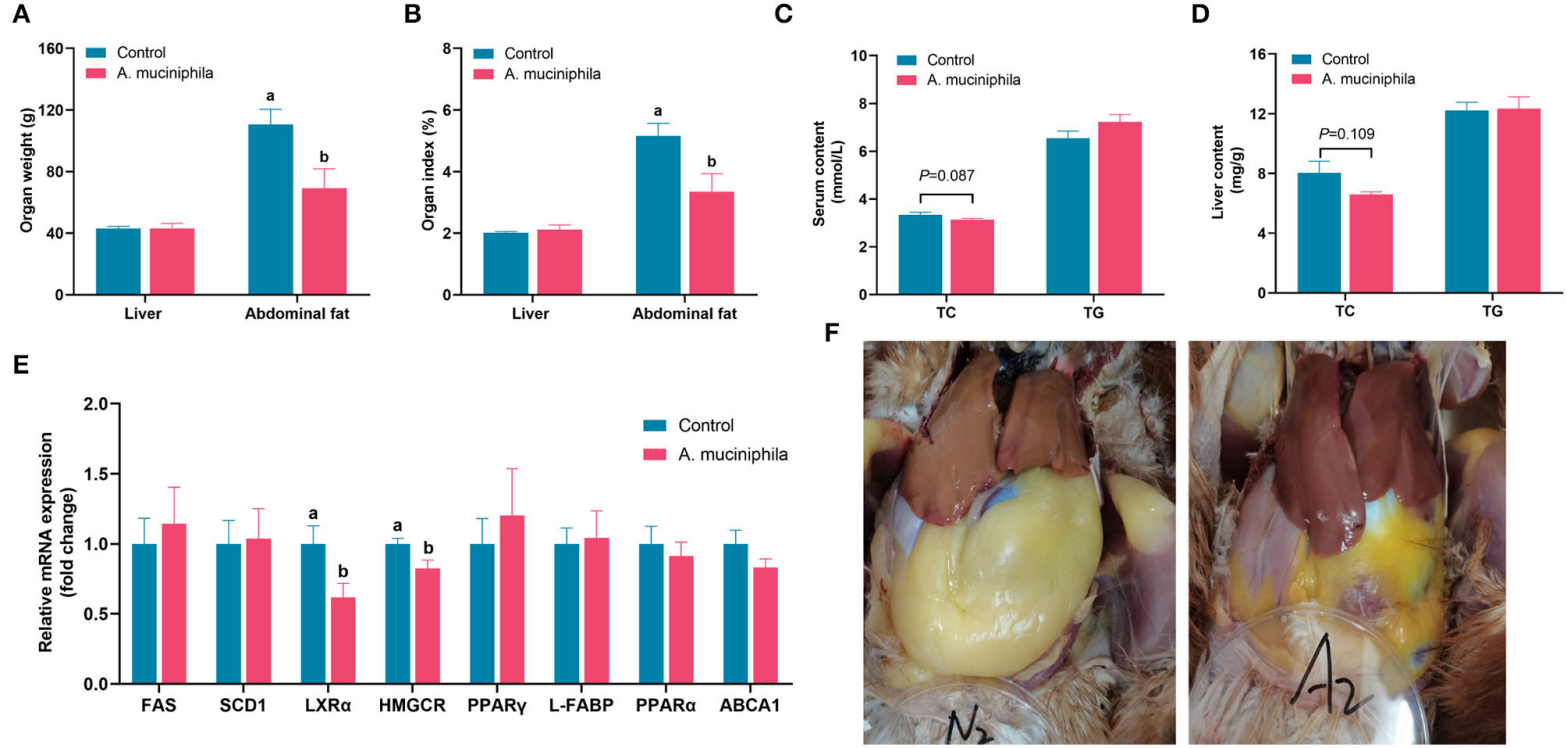
*Akkermansia muciniphila* supplementation ameliorated lipid metabolism in aged laying hens (*n* = 7). **(A)** Liver and abdominal fat weight. **(B)** Liver and abdominal fat index. **(C)** Serum total cholesterol (TC) and triglyceride (TG) levels. **(D)** Hepatic TC and TG levels. **(E)** Relative mRNA expression of lipid metabolism-related genes in the liver. The expression levels of genes in the control group were set as 1 and the relative fold increases were determined by comparison with the control group. **(F)** Diagram representing the anatomy of the liver and abdominal fat. Different letters indicate significant differences at *p* < 0.05. *FAS*, fatty acid synthase; *SCD1*, stearoyl-CoA desaturase 1; *LXR*α, liver X receptor alpha; *HMGCR*, 3-hydroxy-3-methylglutaryl-CoA reductase; *PPAR-*γ, peroxisome proliferator-activated receptor gamma; *L-FABP*, liver fatty acid binding protein; *PPAR-*α, peroxisome proliferator-activated receptor alpha; *ABCA1*, ATP-binding cassette transporter A1.

To explore the beneficial effects of *A. muciniphila* on aged laying hens, which are normally associated with a high incidence of FLHS, we supplemented their diet with *A. muciniphila* lyophilized powder. Interestingly, the addition of lyophilized *A. muciniphila* significantly decreased abdominal fat deposition (*p* < 0.05) (Figures 6A,B), and tended to reduce serum TC levels (*p* = 0.087) (Figure 6C); however, no effect was observed on production performance (egg production rate, average egg weight, average daily feed intake, feed to gain ratio) (Supplementary Table S5), liver weight, or serum and liver TG concentrations (Figures 6A–D). qPCR analysis indicated that treatment with lyophilized powder of *A. muciniphila* did not significantly affect the expression levels of *FAS, SCD1, PPAR-*γ, *L-FABP, PPAR-*α, or *ABCA1*; however, the expression of *LXR*α and *HMGCR* was significantly downregulated in the livers of treated aged hens (*p* < 0.05) (Figure 6E). Furthermore, we found that the livers of hens in the control group were yellowish and lighter than those of animals treated with the lyophilized powder, which displayed a smooth external reddish-brown surface and less abdominal fat deposition (Figure 6F).

### *A. muciniphila* Lyophilized Powder Supplementation Improved Egg Quality in Aged Laying Hens

**Figure 7:**
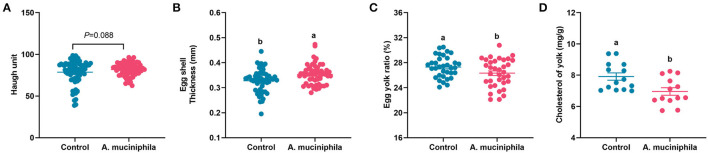
*Akkermansia muciniphila* improved egg quality in aged laying hens. **(A)** Haugh unit (*n* = 63). **(B)** Eggshell thickness (mm) (*n* = 63). **(C)** Egg yolk ratio (%) (*n* = 63). **(D)** Cholesterol content in the yolk (mg/g) (*n* = 14). Different letters indicate significant differences at *p* < 0.05.

The addition of lyophilized powder of *A. muciniphila* led to an increasing trend in the Haugh unit (*p* = 0.088) (Figure 7A), a significant increase in eggshell thickness (*p* < 0.05) (Figure 7B), a lower egg yolk ratio, and reduced yolk cholesterol content (*p* < 0.05) (Figures 7C,D); however, no changes in egg weight, eggshell strength, albumen height, or yolk color were observed (Supplementary Table S6).

### *A. muciniphila* Lyophilized Powder Supplementation Reshaped the Composition of the Gut Microbiota in Aged Laying Hens

**Figure 8:**
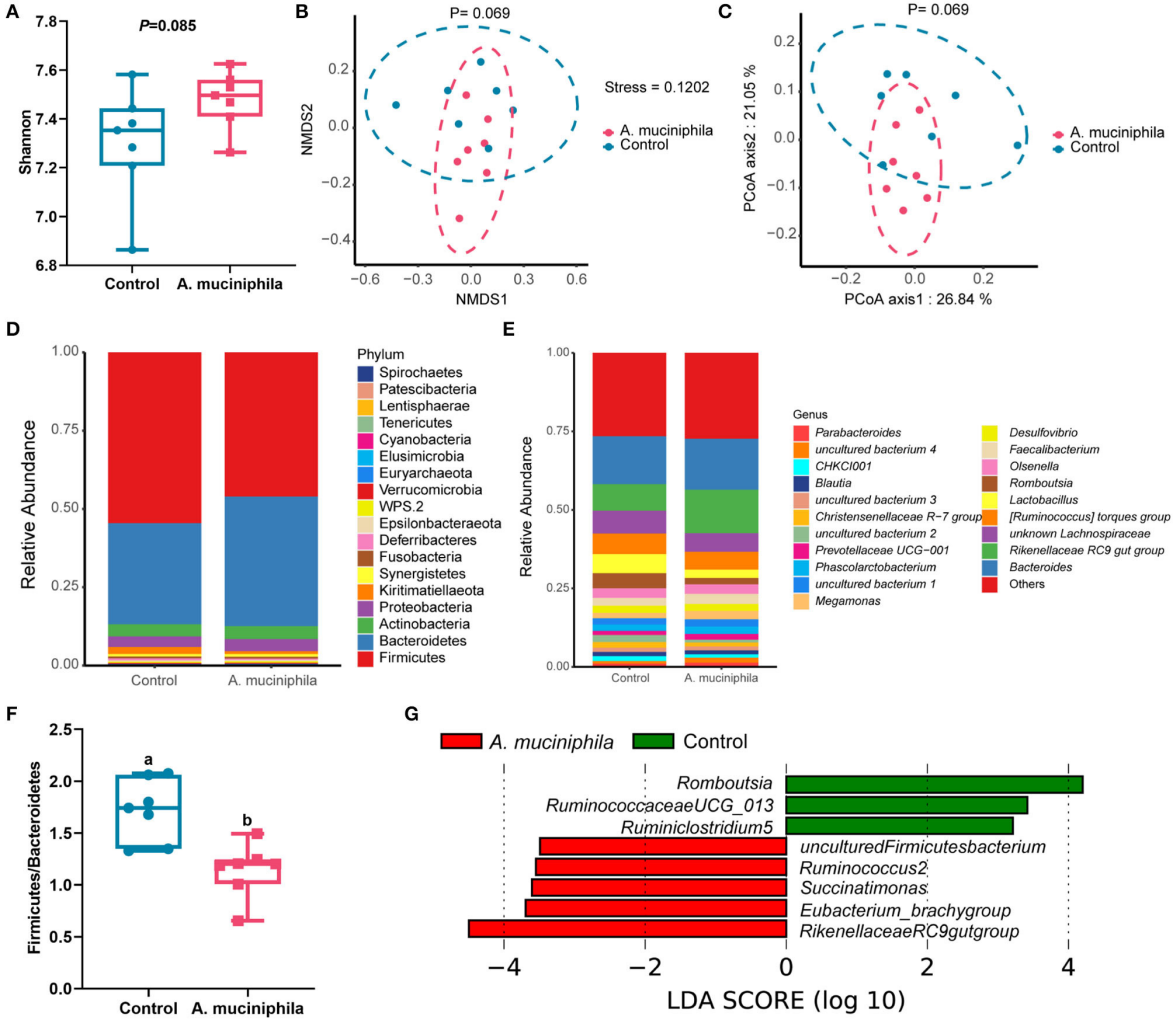
*Akkermansia muciniphila* supplementation reshaped the composition of the gut microbiota in aged laying hens (*n*=7). **(A)** Determination of gut microbiota diversity (as measured by the Shannon index). **(B)** Non-metric multidimensional scaling (NMDS) analysis. **(C)** Principal coordinates analysis (PCoA). **(D)** Taxonomic analysis of the gut microbiota at the phylum level. **(E)** Taxonomic analysis of the gut microbiota at the genus level. **(F)** Ratio of *Firmicutes* to *Bacteroidetes*. **(G)** The linear discriminant analysis (LDA) score showed a significant difference in bacterial composition between the control group and the *A. muciniphila*-treated group. Different letters indicate significant differences at *p* < 0.05.

Alpha-diversity analysis identified a trend toward an increase in gut microbiota diversity (as measured by the Shannon index; *p* = 0.085) (Figure 8A) and separation of microbial communities between the two groups (as determined by both PCoA and NMDS plot analysis) with the addition of lyophilized powder of *A. muciniphila* (*p* = 0.069) (Figures 8B,C). *Firmicutes* and *Bacteroidetes* were the dominant phyla in the cecum of aged laying hens (Figure 8D), and supplementation of lyophilized powder of *A. muciniphila* significantly reduced the ratio of *Firmicutes* to *Bacteroidetes* (*p* < 0.05) (Figure 8F). LEfSe analysis revealed that several beneficial bacteria, such as members of the genera *Ruminococcus*2, *Succinatimonas, Eubacterium_brachy group*, and *Rikenellaceae RC9 gut group*, were significantly enriched (LDA score >2, *p* < 0.05) in the cecum of hens in the *A. muciniphila* lyophilized powder treatment group (Figure 8G).

## Discussion

FLHS is a lipid metabolism disorder disease characterized by pathological hepatic and abdominal fat accumulation. Excessive lipid deposition in the liver is a common pathology in laying hens, and FLHS can develop in adult laying hens during normal aging in the absence of external stimuli (Hamid et al., 2019). In this study, we induced FLHS in laying hens by feeding them a high-energy, low-protein diet, and evaluated the protective effect of *A. muciniphila* against FLHS. The feeding of a high-energy, low-protein diet for 18 weeks significantly increased body weight, abdominal fat deposition, serum TC and TG contents, and liver TG levels in laying hens, which was consistent with previous FLHS modeling results (Song et al., 2017; Yang et al., 2017; Qiu et al., 2021). Several studies have suggested that *A. muciniphila* supplementation can significantly reduce body weight, TC and TG levels in serum and liver, and fat deposition in obese mice (Everard et al., 2013; Zhao et al., 2017; Ashrafian et al., 2019; Wu et al., 2020; Yang et al., 2020). In this study, intervention with either live or pasteurized *A. muciniphila* reduced body weight, serum and liver lipid levels, and abdominal fat deposition in laying hens, indicating that *A. muciniphila* treatment can effectively alleviate FLHS in laying hens. Interestingly, recent studies have shown that pasteurized *A. muciniphila* exerts the same effect on obesity as that seen with live bacteria (Plovier et al., 2017; Depommier et al., 2020); here, we further confirmed the similar effects of the two treatments.

Probiotic strains can exert lipid-lowering activity *via* modulating lipid metabolism-related gene expression (Yoo et al., 2013). *FAS, GPAT, SCD1, LXR*α, and *PPAR-*γ are key enzymes in lipid synthesis. *FAS* plays an important role in catalyzing the elongation of carbon chains of fatty acids (FAs) (Lu et al., 2018), *SCD1* is involved in the regulation of TG synthesis and fatty acid oxidation in the liver (Ntambi et al., 2002; Kotronen et al., 2009; Aijohani et al., 2017), and *GPAT* acts as the rate-limiting enzyme in the synthesis of glycerophospholipids and triacylglycerol (Karasawa et al., 2019). Consistent with previously reported results (Wu W. et al., 2017; Yang et al., 2020), in this study, we found that *FAS, SCD1*, and *GPAT* mRNA expression levels in the liver were reduced following treatment with *A. muciniphila*, concomitant with reduced lipid production in the liver. *LXR*α is a ligand-dependent nuclear receptor that plays a crucial role in *de novo* lipogenesis in the liver (Repa et al., 2000) and the expression of both *FAS* (Joseph et al., 2002) and *SCD1* (Chu et al., 2006) can be directly activated by the binding of LXREs to their promoter regions. Thus, the decreased expression of *LXR*α in the *A. muciniphila* treatment groups suggested that supplementation with this bacterium can inhibit lipid synthesis, likely through the *LXR*α-*FAS*/*SCD1* pathway. *PPAR-*γ has a protective effect on acute liver injury (Tanaka et al., 2017), and the development of hepatic steatosis and fibrosis was reported to be accompanied by the downregulation of *PPAR-*γ expression (Shang et al., 2018). In this study, we found that *A. muciniphila* treatment led to the upregulation of *PPAR-*γ expression, which was in accordance with that reported in a previous study using cell culture (Keshavarz et al., 2021). *CPT1, ACOX1, FXR, PPAR-*α, and *L-FABP* are all involved in lipid transport and oxidation. *CPT1* protein activity represents the rate-limiting step of the carnitine shuttle in the oxidation of long-chain lipids in the mitochondria (Schlaepfer and Joshi, 2020) and *ACOX1* is responsible for the catabolism of very-long-chain FAs (Moreno-Fernandez et al., 2018). Meanwhile, *FXR*, a bile acid nuclear receptor, plays a critical role in regulating bile acid homeostasis and lipid metabolism (Rajani and Jia, 2018). In this study, we demonstrated that the expression of *CPT1, ACOX1*, and *FXR* was increased in the groups fed an HFD, suggesting that FAs decomposition and β-oxidation may be enhanced when hepatic lipid accumulation reaches a certain level. The activation of *FXR* can reduce hepatic lipid levels by regulating *ACOX1* expression through *PPAR-*α (Knottnerus et al., 2018; Rajani and Jia, 2018). However, no differences were detected in the hepatic expression of *CPT1, ACOX1, PPAR-*α, or *FXR* between the HFD group and the *A. muciniphila* groups (HFD_LA and HFD_PA), suggesting that the *A. muciniphila*-induced reduction in liver lipid contents in laying hens with FLHS occurred independently of the *FXR-PPAR*α*-ACOX* pathway. *L-FABP* can promote β-oxidation of medium and long-chain FAs (Hostetler et al., 2011; Schroeder et al., 2016), and *L-FABP* ablation leads to triacylglycerol accumulation in the liver (Lagakos et al., 2011) and hepatocytes (Storey et al., 2012). This suggests that *A. muciniphila* treatment promoted FAs decomposition and inhibited lipid accumulation by upregulating *L-FABP* expression, at least partly.

The gut microbiota are key contributors to the modulation of host lipid metabolism (Lu et al., 2019; Ushiroda et al., 2019) and treatment with probiotics can ameliorate intestinal dysbiosis and reduce obesity by modulating the gut microbiota (Le et al., 2019). In the present study, *A. muciniphila* treatment did not significantly affect cecal microbial richness and diversity; however, supplementation with this bacterium exerted anti-FLHS effects by increasing the abundance of microorganisms inversely associated with obesity, as well as promoting the growth of acid-producing microorganisms in the gut.

The Clostridia are major producers of SCFAs (Louis et al., 2010) and their abundance is decreased in the gut of obese mice (Monk et al., 2019; Zhao et al., 2019). Most of the microbial species enriched in the *A. muciniphila* groups (such as *Ruminococcus*2, *Blautia, Clostridium* sp. CAG:306, and *Clostridium sensu stricto* 1) belong to the *Clostridia* class. In addition, the abundance of *Intestinimonas* (Thingholm et al., 2019) and *Phascolarctobacterium* (Zhang et al., 2015; Wu F. et al., 2017) was reported to be significantly reduced in obesity, and *Enterococcus* treatment resulted in reduced body weight and serum lipid levels in rats fed an HFD (Zhang et al., 2017). Here, we found that *A. muciniphila* treatment increased the abundances of *Clostridia, Intestinimonas, Phascolarctobacterium*, and *Enterococcus*, which may be beneficial for decreasing lipid deposition in the liver of laying hens with FLHS.

Dietary nutrients and microbial-derived SCFAs in the intestinal tract are absorbed *via* intestinal epithelial cells and travel through the portal vein to the liver for metabolism (Zhang et al., 2021). A significant reduction in SCFAs-producing bacteria was noted in patients with hepatic steatosis (Smirnova et al., 2020). SCFAs supplementation can improve host lipid metabolism (Besten et al., 2015) and promote lipid oxidation (Beek et al., 2016; Canfora et al., 2017). These findings suggest that SCFAs have protective effects against NAFLD. *A. muciniphila* resides in the mucus layer and metabolizes mucus, producing both acetate and propionate (Belzer and de Vos, 2012). The generated propionate can act on intestinal tissue through the SCFAs receptor *GPR43*, while other SCFAs can also signal to the host through *GPR41* (Derrien et al., 2011), and are involved in the regulation of lipid metabolism (Nøhr et al., 2013). The product of *A. muciniphila*-mediated mucin decomposition can also be used as raw material for other SCFAs-producing bacteria (Belzer et al., 2017). Our results showed that the SCFA content was significantly increased in the cecum of *A. muciniphila*-treated laying hens, while *Flavonifractor, Faecalitalea*, and *Enterococcus* became the dominant bacteria in treated animals. *Intestinimonas* (Bui et al., 2020), *Flavonifractor* (Meng et al., 2019), *Faecalitalea* (Eeckhaut et al., 2011), and *Enterococcus* (Allameh et al., 2017) are important propionate and butyrate producers. Here, we found that these microorganisms were significantly and positively correlated with the contents of most SCFAs, while *A. muciniphila* administration upregulated the expression of both *GPR43* and *GPR41* in the liver. The above results collectively indicated that *A. muciniphila* can promote the growth of SCFAs-producing microorganisms in the intestinal tract of laying hens, leading to increased SCFAs production and the subsequent alleviation of FLHS symptoms, likely through signaling *via* the SCFA receptors in the liver.

Dietary probiotic supplementation can improve egg production (Yörük et al., 2004) and quality (Zhang et al., 2013). Albumen height and the Haugh unit are important indicators of the internal quality of eggs. The higher the value of the Haugh unit, the better the consistency and the higher the quality of the egg white. In this study, *A. muciniphila* treatment improved egg quality, as determined through an observed increase in the Haugh unit value. *A. muciniphila* also reduced the cholesterol content in the egg yolk, which may be related to the ability of *A. muciniphila* to reduce blood and liver lipid concentrations. Eggs have long been considered a nutrient-dense food owing to their high content of bioactive lipids, which are found almost exclusively in the yolk (Xiao et al., 2020). To better understand the effects of *A. muciniphila* intervention on the egg yolk lipid profile, we undertook a lipidomics analysis using LC–Q–TOF–MS. TGs, PCs, and PEs were the most abundant lipid species identified in ES+ mode, while PEs and PCs were the most abundant lipid species detected in ES– mode, which is consistent with previous findings (Yu et al., 2019a,b). We found a clear separation between the ND and HFD groups in both the ES+ and ES– patterns, suggesting that diet was the main contributor to the altered egg yolk lipid profiles. PCs are the main components of the cell membrane and participate in a variety of biological processes (Ming et al., 2017; Veen et al., 2017). Dietary PC supplementation can improve blood lipid levels (Jan et al., 2017), alleviate hepatic steatosis (Yanagita, 2005), and reduce hepatic lipid deposition (Yu et al., 2020). Dietary PE can reduce plasma TG and TC concentrations (Wilson et al., 1998). In this study, most PC and PE species were downregulated in the HFD group relative to the ND group, which may be related to the FLHS induced by the high-energy, low-protein diet. The reduction in the TG content and the increase in that of PE in the egg yolk after treatment with live *A. muciniphila* may have potentially beneficial effects on obesity. High-fat diet-induced obesity is closely related to dietary TGs, especially long-chain ones (LCTs) (Poret et al., 2018). Studies on humans have shown that, compared with medium-chain TGs (MCTs), chronic ingestion of LCTs reduces fat oxidation and energy expenditure and increases hepatic TG levels (St-Onge et al., 2014), which subsequently leads to greater fat accumulation (St-Onge and Jones, 2003). In this study, a total of 54 TG metabolites were detected in the HFD and HFD_PA groups. Most of the upregulated TGs in the HFD group were LCTs, while most of the TGs showing an increased abundance in the HFD_PA group were MCTs. Additionally, 11 diglycerides (DGs) were also upregulated in the HFD_PA group. Dietary DGs can exert anti-obesity effects by reducing body weight and visceral fat mass (Hue et al., 2009). These results relating to differential lipid profile in egg yolks suggested that *A. muciniphila* treatment can improve the nutritional value and health benefits of eggs by serving as a potentially beneficial lipid resource in the diet. However, the effects of live and pasteurized *A. muciniphila* on the lipid profile of egg yolks were not identical, and understanding why requires further investigation.

Given that older laying hens have a higher incidence of FLHS than younger ones (Shini et al., 2019), therefore, we also evaluated the potential benefits of *A. muciniphila* in hen production and health through the supplementation of *A. muciniphila* lyophilized powder in the diets of aged laying hens. The results showed that the addition of *A. muciniphila* lyophilized powder could significantly reduce abdominal fat deposition in aged laying hens, and there was a trend toward a lower serum TC content, indicating that *A. muciniphila* exerted a similar ameliorative effect on lipid metabolism in aged laying hens as that seen in laying hens with HFD-induced FLHS. However, unlike in the latter, *A. muciniphila* mainly downregulated the mRNA expression of *HMGCR*, encoding the rate-limiting enzyme in the hepatic cholesterol biosynthesis pathway (Trapani et al., 2011), and *LXR*α in the liver of aged laying hens, which resulted in reduced cholesterol and lipid accumulation. In addition, dietary *A. muciniphila* significantly increased eggshell thickness and reduced the egg yolk ratio in aged laying hens—effects that were not seen in laying hens with FLHS.

*A. muciniphila* supplementation also reshaped the composition of the gut microbiota in aged laying hens. Studies have consistently shown that the abundance of *Bacterioidetes* is decreased and that of *Firmicutes* increased in the gut of obese patients (Ley et al., 2006; Bervoets et al., 2013). Moreover, changes in the *Bacterioidetes*/*Firmicutes* ratio can affect energy harvesting (Fernandes et al., 2014), leading to lipid accumulation and the development of a fatty liver (Fontaine et al., 2019; Ushiroda et al., 2019). In our study, the increase in microbial diversity, the reduction of the *Firmicutes*/*Bacteroidetes* ratio, and the increase in the abundance of *Ruminococcus2*, which is inversely associated with obesity, resulting from *A. muciniphila* treatment may have contributed to the decline in lipid deposition in laying hens. However, *A. muciniphila* exerted different effects on lipid metabolism, gut microbiota composition, and egg quality between laying hens with FLHS and aged laying hens, which may be related to differences in factors such as lipid metabolism, gut flora structure, and egg physical parameters at different stages of laying hen development.

## Conclusion

*A. muciniphila* intervention reduced lipid deposition, altered gut microbiota composition, and improved egg quality and egg yolk lipid profiles both in laying hens with FLHS and aged laying hens, indicating that *A. muciniphila* can modulate lipid metabolism and thereby promote laying hen health as well as egg quality and nutritive value. Live, pasteurized, and lyophilized *A. muciniphila* preparations all have the potential for use as additives for improving laying hen production.

## Data Availability Statement

The datasets generated for this study can be found in the Sequence Read Archive of the NCBI (Accession No. PRJNA831016).

## Ethics Statement

The animal study was reviewed and approved by China Agricultural University Animal Care and Use Committee. Written informed consent was obtained from the owners for the participation of their animals in this study.

## Author Contributions

The research was mainly conceived and designed by DL and FW and conducted by FW, XY, and CX. FW and MZ analyzed the data. FW wrote the manuscript. XY, CX, and MZ contributed to sample collection and analysis. YH and DL critically reviewed the manuscript. All authors read and approved the final manuscript.

## Funding

This work was supported by the National Natural Science Foundation of China (Grant No. 32172685).

## Conflict of Interest

DL, YH, and FW hold a Chinese patent for the application of *Akkermansia muciniphila* in laying hens: Patent number 202210208096.5. The remaining authors declare that the research was conducted in the absence of any commercial or financial relationships that could be construed as a potential conflict of interest.

## Publisher's Note

All claims expressed in this article are solely those of the authors and do not necessarily represent those of their affiliated organizations, or those of the publisher, the editors and the reviewers. Any product that may be evaluated in this article, or claim that may be made by its manufacturer, is not guaranteed or endorsed by the publisher.

## References

[B1] Aijohani A. L. Syed D. N. Ntambi J. M. (2017). Insights into stearoyl-CoA desaturase-1 regulation of systemic metabolism. Trends Endocrinol. Metab. 28, 831–842. 10.1016/j.tem.2017.10.003 29089222 PMC5701860

[B2] Allameh S. K. Ring,ø E. Yusoff F. M. Daud H. M. Ideris A. (2017). Dietary supplement ofEnterococcus faecalison digestive enzyme activities, short-chain fatty acid production, immune system response and disease resistance of Javanese carp (Puntius gonionotus,Bleeker 1850). Aquac Nutr. 23, 331–338. 10.1111/anu.12397

[B3] Arab J. P. Martin-Mateos R. M. Shah V. H. (2018). Gut-liver axis, cirrhosis and portal hypertension: the chicken and the egg. Hepatol. Int. 12, 24–33. 10.1007/s12072-017-9798-x 28550391 PMC6876989

[B4] Ashrafian F. Shahriary A. Behrouzi A. Moradi H. R. Keshavarz Azizi Raftar S. Lari A.. (2019). Akkermansia muciniphila-derived extracellular vesicles as a mucosal delivery vector for amelioration of obesity in mice. Front. Microbiol. 10, 2155. 10.3389/fmicb.2019.02155 31632356 PMC6779730

[B5] Beek C. M. V. D. Canfora E. E. Lenaerts K. Troost F. J. Damink S. Holst J. J.. (2016). Distal, not proximal, colonic acetate infusions promote fat oxidation and improve metabolic markers in overweight/obese men. Clin. Sci. 130, 2073–2082. 10.1042/CS20160263 27439969

[B6] Belzer C. Chia L. W. Aalvink S. Chamlagain B. Piironen V. Knol J.. (2017). Microbial metabolic networks at the mucus layer lead to diet-independent butyrate and vitamin B(12) production by intestinal symbionts. mBio. 8. 10.1128/mBio.00770-17 28928206 PMC5605934

[B7] Belzer C. de Vos W. M. (2012). Microbes inside–from diversity to function: the case of Akkermansia. ISME J. 6, 1449–1458. 10.1038/ismej.2012.6 22437156 PMC3401025

[B8] Bervoets L. Van Hoorenbeeck K. Kortleven I. Van Noten C. Hens N. Vael C.. (2013). Differences in gut microbiota composition between obese and lean children: a cross-sectional study. Gut Pathog. 5, 10. 10.1186/1757-4749-5-10 23631345 PMC3658928

[B9] Besten G. D. Bleeker A. Gerding A. Eunen K. V. Havinga R. Dijk T. H. V.. (2015). Short-chain fatty acids protect against high-fat diet-induced obesity via a pparγ-dependent switch from lipogenesis to fat oxidation. Diabetes. 64, 2398–2408. 10.2337/db14-1213 25695945

[B10] Boursier J. Diehl A. M. (2016). Nonalcoholic fatty liver disease and the gut microbiome. Clin. Liver Dis. 263–75. 10.1016/j.cld.2015.10.012 27063268

[B11] Bui T. P. N. Troise A. D. Nijsse B. Roviello G. N. Fogliano V. Vos W. M. (2020). Intestinimonas-like bacteria are important butyrate producers that utilize Nε-fructosyllysine and lysine in formula-fed infants and adults. J. Funct. Foods. 70. 10.1016/j.jff.2020.103974

[B12] Calik A. Ergün A. (2015). Effect of lactulose supplementation on growth performance, intestinal histomorphology, cecal microbial population, and short-chain fatty acid composition of broiler chickens. Poult. Sci. 94, 2173–2182. 10.3382/ps/pev182 26188035

[B13] Canfora E. E. Beek C. M. V. D. Jocken J. W. E. Goossens G. H. Holst J. J. Olde-Damink S. W. M.. (2017). Colonic infusions of short-chain fatty acid mixtures promote energy metabolism in overweight/obese men: a randomized crossover trial. Sci. Rep. 7, 2360. 10.1038/s41598-017-02546-x 28539646 PMC5443817

[B14] Choi Y. I. Ahn H. J. Lee B. K. Oh S. T. An B. K. Kang C. W. (2012). Nutritional and hormonal induction of fatty liver syndrome and effects of dietary lipotropic factors in egg-type male chicks. Asian-australas. J. Anim. Sci. 25, 1145–1152. 10.5713/ajas.2011.11418 25049674 PMC4092996

[B15] Chu K. Miyazaki M. Man W. C. Ntambi J. M. (2006). Stearoyl-coenzyme A desaturase 1 deficiency protects against hypertriglyceridemia and increases plasma high-density lipoprotein cholesterol induced by liver X receptor activation. Mol. Cell. Biol. 26, 6786–6798. 10.1128/MCB.00077-06 16943421 PMC1592860

[B16] Cole B. K. Feaver R. E. Wamhoff B. R. Dash A. (2018). Non-alcoholic fatty liver disease (NAFLD) models in drug discovery. Expert Opin. Drug Discov. 13, 193–205. 10.1080/17460441.2018.1410135 29190166

[B17] Cui L. Decker E. A. (2016). Phospholipids in foods: prooxidants or antioxidants? J. Sci. Food Agric. 96, 18–31. 10.1002/jsfa.7320 26108454

[B18] Dao M. C. Everard A. Aron-Wisnewsky J. Sokolovska N. Prifti E. Verger E. O.. (2016). Akkermansia muciniphila and improved metabolic health during a dietary intervention in obesity: relationship with gut microbiome richness and ecology. Gut. 65, 426–436. 10.1136/gutjnl-2014-308778 26100928

[B19] Depommier C. Van Hul M. Everard A. Delzenne N. M. De Vos W. M. Cani P. D. (2020). Pasteurized Akkermansia muciniphila increases whole-body energy expenditure and fecal energy excretion in diet-induced obese mice. Gut Microbes. 11, 1231–1245. 10.1080/19490976.2020.1737307 32167023 PMC7524283

[B20] Derrien M. Baarlen P. V. Hooiveld G. Norin E. Müller M. Vos W. M. (2011). Modulation of mucosal immune response, tolerance, and proliferation in mice colonized by the mucin-degrader akkermansia muciniphila. Front. Microbiol. 2, 166. 10.3389/fmicb.2011.00166 21904534 PMC3153965

[B21] Eeckhaut V. Van Immerseel F. Croubels S. De Baere S. Haesebrouck F. Ducatelle R.. (2011). Butyrate production in phylogenetically diverse Firmicutes isolated from the chicken caecum. Microb. Biotechnol. 4, 503–512. 10.1111/j.1751-7915.2010.00244.x 21375722 PMC3815262

[B22] Everard A. Belzer C. Geurts L. Ouwerkerk J. P. Druart C. Bindels L. B.. (2013). Cross-talk between Akkermansia muciniphila and intestinal epithelium controls diet-induced obesity. P Natl Acad Sci USA. 110, 9066–9071. 10.1073/pnas.1219451110 23671105 PMC3670398

[B23] Fernandes J. Su W. Rahat-Rozenbloom S. Wolever T. M. Comelli E. M. (2014). Adiposity, gut microbiota and faecal short chain fatty acids are linked in adult humans. Nutr. Diabetes. 4, e121. 10.1038/nutd.2014.23 24979150 PMC4079931

[B24] Folch J. (1957). A simple method for the isolation and purification of total lipids from animal tissues. J. Biol. Chem. 226. 10.1016/S0021-9258(18)64849-5 13428781

[B25] Fontaine M. A. Diane A. Singh V. P. Mangat R. Krysa J. A. Nelson R.. (2019). Low birth weight causes insulin resistance and aberrant intestinal lipid metabolism independent of microbiota abundance in Landrace-Large White pigs. FASEB J. 33, 9250–9262. 10.1096/fj.201801302RR 31144992

[B26] Hamid H. Zhang J. Y. Li W. X. Liu C. Li M. L. Zhao L. H.. (2019). Interactions between the cecal microbiota and non-alcoholic steatohepatitis using laying hens as the model. Poult. Sci. 98, 2509–2521. 10.3382/ps/pey596 30690636

[B27] Hammad S. M. Siegel H. S. Marks H. L. (1996). Dietary cholesterol effects on plasma and yolk cholesterol fractions in selected lines of Japanese quail. Poult. Sci. 75, 933–942. 10.3382/ps.0750933 8966183

[B28] Hostetler H. A. Lupas D. Tan Y. Dai J. Kelzer M. S. Martin G. G.. (2011). Acyl-CoA binding proteins interact with the acyl-CoA binding domain of mitochondrial carnitine palmitoyl transferase I. Mol. Cell. Biochem. 355, 135–148. 10.1007/s11010-011-0847-9 21541677 PMC3149718

[B29] Hue J. J. Lee K. N. Jeong J. H. Lee S. H. Lee Y. H. Jeong S. W.. (2009). Anti-obesity activity of diglyceride containing conjugated linoleic acid in C57BL/6J ob/ob mice. J. Vet. Sci. 10, 189–195. 10.4142/jvs.2009.10.3.189 19687618 PMC2801123

[B30] Jan M. Thondre P. S. El-Chab A. Lightowler H. J. (2017). The effect of dietary phosphatidylcholine supplementation on lipid profile in mild hyperlipidaemic individuals. Proc. Nutr. Soc. 76, E219. 10.1017/S0029665117003810

[B31] Joseph S. B. Laffitte B. A. Patel P. H. Watson M. A. Matsukuma K. E. Walczak R.. (2002). Direct and indirect mechanisms for regulation of fatty acid synthase gene expression by liver X receptors. J. Biol. Chem. 277, 11019–11025. 10.1074/jbc.M111041200 11790787

[B32] Karasawa K. Tanigawa K. Harada A. Yamashita A. (2019). Transcriptional regulation of acyl-CoA:glycerol-sn-3-phosphate acyltransferases. Int. J. Mol. Sci. 20. 10.3390/ijms20040964 30813330 PMC6412627

[B33] Karlsson C. L. Onnerfält J. Xu J. Molin G. Ahrn,é S. Thorngren-Jerneck K. (2012). The microbiota of the gut in preschool children with normal and excessive body weight. Obesity (Silver Spring, Md). 20, 2257–2261. 10.1038/oby.2012.110 22546742

[B34] Keshavarz A. R. S. Abdollahiyan S. Azimirad M. Yadegar A. Vaziri F. Moshiri A.. (2021). The anti-fibrotic effects of heat-killed akkermansia muciniphila muct on liver fibrosis markers and activation of hepatic stellate cells. Probiotics Antimicrob. Proteins. 13, 776–787. 10.1007/s12602-020-09733-9 33433897

[B35] Knottnerus S. J. G. Bleeker J. C. Wüst R. C. I. Ferdinandusse S. L I. J. Wijburg F. A.. (2018). Disorders of mitochondrial long-chain fatty acid oxidation and the carnitine shuttle. Rev Endocr Metab Dis. 19, 93–106. 10.1007/s11154-018-9448-1 29926323 PMC6208583

[B36] Kotronen A. Seppänen-Laakso T. Westerbacka J. Kiviluoto T. Arola J. Ruskeep,ää A. L.. (2009). Hepatic stearoyl-CoA desaturase (SCD)-1 activity and diacylglycerol but not ceramide concentrations are increased in the nonalcoholic human fatty liver. Diabetes. 58, 203–208. 10.2337/db08-1074 18952834 PMC2606873

[B37] Lagakos W. S. Gajda A. M. Agellon L. Binas B. Choi V. Mandap B.. (2011). Different functions of intestinal and liver-type fatty acid-binding proteins in intestine and in whole body energy homeostasis. Am. J. Physiol. Gastrointest. Liver Physiol. 300, G803–G814. 10.1152/ajpgi.00229.2010 21350192 PMC3094135

[B38] Le B. M. Daniel N. Varin T. V. Naimi S. Demers-Mathieu V. Pilon G.. (2019). In vivo screening of multiple bacterial strains identifies Lactobacillus rhamnosus Lb102 and Bifidobacterium animalis ssp. lactis Bf141 as probiotics that improve metabolic disorders in a mouse model of obesity. FASEB J. 33, 4921–35. 10.1096/fj.201801672R 30596521

[B39] Ley R. E. Turnbaugh P. J. Klein S. Gordon J. I. (2006). Microbial ecology: human gut microbes associated with obesity. Nature. 444, 1022–1023. 10.1038/4441022a 17183309

[B40] Louis P. Young P. Holtrop G. Flint H. J. (2010). Diversity of human colonic butyrate-producing bacteria revealed by analysis of the butyryl-CoA:acetate CoA-transferase gene. Environ. Microbiol. 12, 304–314. 10.1111/j.1462-2920.2009.02066.x 19807780

[B41] Lu M. Cao Y. Xiao J. Song M. Ho C. T. (2018). Molecular mechanisms of the anti-obesity effect of bioactive ingredients in common spices: a review. Food Funct. 9, 4569–4581. 10.1039/C8FO01349G 30168574

[B42] Lu X. Liu J. Zhang N. Fu Y. Zhang Z. Li Y.. (2019). Ripened Pu-erh tea extract protects mice from obesity by modulating gut microbiota composition. J. Agric. Food Chem. 67, 6978–6994. 10.1021/acs.jafc.8b04909 31070363

[B43] Meng Q. Sun S. Luo Z. Shi B. Shan A. Cheng B. (2019). Maternal dietary resveratrol alleviates weaning-associated diarrhea and intestinal inflammation in pig offspring by changing intestinal gene expression and microbiota. Food Funct. 10, 5626–5643. 10.1039/C9FO00637K 31432838

[B44] Ming Y. N. Zhang J. Y. Wang X. L. Li C. M. Ma S. C. Wang Z. Y.. (2017). Liquid chromatography mass spectrometry-based profiling of phosphatidylcholine and phosphatidylethanolamine in the plasma and liver of acetaminophen-induced liver injured mice. Lipids Health Dis. 16, 153. 10.1186/s12944-017-0540-4 28807032 PMC5556666

[B45] Monk J. M. Wu W. Lepp D. Wellings H. R. Hutchinson A. L. Liddle D. M.. (2019). Navy bean supplemented high-fat diet improves intestinal health, epithelial barrier integrity and critical aspects of the obese inflammatory phenotype. J. Nutr. Biochem. 70, 91–104. 10.1016/j.jnutbio.2019.04.009 31195365

[B46] Moreno-Fernandez M. E. Giles D. A. Stankiewicz T. E. Sheridan R. Karns R. Cappelletti M.. (2018). Peroxisomal beta-oxidation regulates whole body metabolism, inflammatory vigor, and pathogenesis of nonalcoholic fatty liver disease. JCI Insight. 3. 10.1172/jci.insight.93626 29563328 PMC5926941

[B47] Natividad J. M. Lamas B. Pham H. P. Michel M. L. Rainteau D. Bridonneau C.. (2018). Bilophila wadsworthia aggravates high fat diet induced metabolic dysfunctions in mice. Nat. Commun. 9, 2802. 10.1038/s41467-018-05249-7 30022049 PMC6052103

[B48] Nøhr M. K. Pedersen M. H. Gille A. Egerod K. L. Engelstoft M. S. Husted A. S.. (2013). GPR41/FFAR3 and GPR43/FFAR2 as cosensors for short-chain fatty acids in enteroendocrine cells vs FFAR3 in enteric neurons and FFAR2 in enteric leukocytes. Endocrinology. 154, 3552–3564. 10.1210/en.2013-1142 23885020

[B49] Ntambi J. M. Miyazaki M. Stoehr J. P. Lan H. Kendziorski C. M. Yandell B. S.. (2002). Loss of stearoyl-CoA desaturase-1 function protects mice against adiposity. PNAS. 99, 11482–11486. 10.1073/pnas.132384699 12177411 PMC123282

[B50] Plovier H. Everard A. Druart C. Depommier C. Hul M. V. Geurts L.. (2017). A purified membrane protein from Akkermansia muciniphila or the pasteurized bacterium improves metabolism in obese and diabetic mice. Nat. Med. 23, 107–113. 10.1038/nm.4236 27892954

[B51] Poret J. M. Souza-Smith F. Marcell S. J. Gaudet D. A. Tzeng T. H. Braymer H. D.. (2018). High fat diet consumption differentially affects adipose tissue inflammation and adipocyte size in obesity-prone and obesity-resistant rats. Int. Obes (Lond). 42, 535–541. 10.1038/ijo.2017.280 29151595 PMC5876080

[B52] Qiu K. Zhao Q. Wang J. Qi G. H. Wu S. G. Zhang H. J. (2021). Effects of pyrroloquinoline quinone on lipid metabolism and anti-oxidative capacity in a high-fat-diet metabolic dysfunction-associated fatty liver disease chick model. Int. J. Mol. Sci. 22. 10.3390/ijms22031458 33535680 PMC7867196

[B53] Rajani C. Jia W. (2018). Bile acids and their effects on diabetes. Front. Med. 12, 608–623. 10.1007/s11684-018-0644-x 30306382

[B54] Repa J. J. Liang G. Ou J. Bashmakov Y. Lobaccaro J. M. Shimomura I.. (2000). Regulation of mouse sterol regulatory element-binding protein-1c gene (SREBP-1c) by oxysterol receptors, LXR alpha and LXR beta. Gene Dev. 14, 2819–2830. 10.1101/gad.844900 11090130 PMC317055

[B55] Reunanen J. Kainulainen V. Huuskonen L. Ottman N. Belzer C. Huhtinen H.. (2015). Akkermansia muciniphila adheres to enterocytes and strengthens the integrity of the epithelial cell layer. Appl. Environ. Microbiol. 81, 3655–3662. 10.1128/AEM.04050-14 25795669 PMC4421065

[B56] Schlaepfer I. R. Joshi M. (2020). CPT1A-mediated fat oxidation, mechanisms, and therapeutic potential. Endocrinology. 161. 10.1210/endocr/bqz046 31900483

[B57] Schneeberger M. Everard A. Gómez-Valadés A. G. Matamoros S. Ramírez S. Delzenne N. M.. (2015). Akkermansia muciniphila inversely correlates with the onset of inflammation, altered adipose tissue metabolism and metabolic disorders during obesity in mice. Sci. Rep. 5, 16643. 10.1038/srep16643 26563823 PMC4643218

[B58] Schroeder F. McIntosh A. L. Martin G. G. Huang H. Landrock D. Chung S.. (2016). Fatty acid binding protein-1 (FABP1) and the human FABP1 T94A variant: roles in the endocannabinoid system and dyslipidemias. Lipids. 51, 655–676. 10.1007/s11745-016-4155-8 27117865 PMC5408584

[B59] Shang L. Hosseini M. Liu X. Kisseleva T. Brenner D. A. (2018). Human hepatic stellate cell isolation and characterization. J. Gastroenterol. 53, 6–17. 10.1007/s00535-017-1404-4 29094206

[B60] Shi Z. Lei H. Chen G. Yuan P. Cao Z. Ser H. L.. (2021). Impaired intestinal akkermansia muciniphila and aryl hydrocarbon receptor ligands contribute to nonalcoholic fatty liver disease in mice. mSystems. 6. 10.1128/mSystems.00985-20 33622853 PMC8573958

[B61] Shini A. Shini S. Bryden W. L. (2019). Fatty liver haemorrhagic syndrome occurrence in laying hens: impact of production system. Avian Pathol. 48, 25–34. 10.1080/03079457.2018.1538550 30345810

[B62] Skórkowska-Telichowska K. Kosińska J. Chwojnicka M. Tuchendler D. Tabin M. Tuchendler R.. (2016). Positive effects of egg-derived phospholipids in patients with metabolic syndrome. Adv Med Sci-poland. 61, 169–174. 10.1016/j.advms.2015.12.003 26829066

[B63] Smirnova E. Puri P. Muthiah M. D. Daitya K. Brown R. Chalasani N.. (2020). Fecal microbiome distinguishes alcohol consumption from alcoholic hepatitis but does not discriminate disease severity. Hepatology (Baltimore, Md). 72, 271–286. 10.1002/hep.31178 32056227 PMC7752764

[B64] Song Y. Ruan J. Luo J. Wang T. Yang F. Cao H.. (2017). Abnormal histopathology, fat percent and hepatic apolipoprotein A I and apolipoprotein B100 mRNA expression in fatty liver hemorrhagic syndrome and their improvement by soybean lecithin. Poult. Sci. 96, 3559–3563. 10.3382/ps/pex163 28938763

[B65] St-Onge M. P. Jones P. J. (2003). Greater rise in fat oxidation with medium-chain triglyceride consumption relative to long-chain triglyceride is associated with lower initial body weight and greater loss of subcutaneous adipose tissue. Int. J. Obes. Relat. Metab. Dis. 27, 1565–1571. 10.1038/sj.ijo.0802467 12975635

[B66] St-Onge M. P. Mayrsohn B. O'Keeffe M. Kissileff H. R. Choudhury A. R. Laferrère B. (2014). Impact of medium and long chain triglycerides consumption on appetite and food intake in overweight men. Eur. J. Clin. Nutr. 68, 1134–1140. 10.1038/ejcn.2014.145 25074387 PMC4192077

[B67] Storey S. M. McIntosh A. L. Huang H. Martin G. G. Landrock K. K. Landrock D.. (2012). Loss of intracellular lipid binding proteins differentially impacts saturated fatty acid uptake and nuclear targeting in mouse hepatocytes. Am. J. Physiol. Gastrointest. Liver Physiol. 303, G837–G850. 10.1152/ajpgi.00489.2011 22859366 PMC3469595

[B68] Tanaka N. Aoyama T. Kimura S. Gonzalez F. J. (2017). Targeting nuclear receptors for the treatment of fatty liver disease. Pharmacol. Ther. 179, 142–157. 10.1016/j.pharmthera.2017.05.011 28546081 PMC6659998

[B69] Thingholm L. B. Ruhlemann M. C. Koch M. Fuqua B. Laucke G. Boehm R.. (2019). Obese individuals with and without type 2 diabetes show different gut microbial functional capacity and composition. Cell Host Microbe. 26, 252-64 e10. 10.1016/j.chom.2019.07.004 31399369 PMC7720933

[B70] Trapani L. Segatto M. Simeoni V. Balducci V. Dhawan A. Parmar V. S.. (2011). Short- and long-term regulation of 3-hydroxy 3-methylglutaryl coenzyme A reductase by a 4-methylcoumarin. Biochimie. 93, 1165–1171. 10.1016/j.biochi.2011.04.009 21530605

[B71] Tumová E. Uhlírov,á L. Tuma R. Chodov,á D. Máchal L. (2017). Age related changes in laying pattern and egg weight of different laying hen genotypes. Anim Prod Sci. 183, 21–26. 10.1016/j.anireprosci.2017.06.006 28683955

[B72] Ushiroda C. Naito Y. Takagi T. Uchiyama K. Mizushima K. Higashimura Y.. (2019). Green tea polyphenol (epigallocatechin-3-gallate) improves gut dysbiosis and serum bile acids dysregulation in high-fat diet-fed mice. J. Clin. Biochem. Nutr. 65, 34–46. 10.3164/jcbn.18-116 31379412 PMC6667385

[B73] Veen J. N. Kennelly J. P. Wan S. Vance J. E. Vance D. E. Jacobs R. L. (2017). The critical role of phosphatidylcholine and phosphatidylethanolamine metabolism in health and disease. Biochim Biophys Acta Biomembr. 1859, 1558–1572. 10.1016/j.bbamem.2017.04.006 28411170

[B74] Wilson T. A. Meservey C. M. Nicolosi R. J. (1998). Soy lecithin reduces plasma lipoprotein cholesterol and early atherogenesis in hypercholesterolemic monkeys and hamsters: beyond linoleate. Atherosclerosis. 140, 147–153. 10.1016/S0021-9150(98)00132-4 9733225

[B75] Wu F. Guo X. Zhang J. Zhang M. Ou Z. Peng Y. (2017). Phascolarctobacterium faecium abundant colonization in human gastrointestinal tract. Exp. Ther. Med. 14, 3122–3126. 10.3892/etm.2017.4878 28912861 PMC5585883

[B76] Wu F. Guo X. Zhang M. Ou Z. Wu D. Deng L.. (2020). An Akkermansia muciniphila subtype alleviates high-fat diet-induced metabolic disorders and inhibits the neurodegenerative process in mice. Anaerobe. 61, 102138. 10.1016/j.anaerobe.2019.102138 31830598

[B77] Wu Q. Tang H. Wang H. (2019). The anti-oxidation and mechanism of essential oil of paederia scandens in the NAFLD model of chicken. Animals. 9, 850. 10.3390/ani9100850 31652524 PMC6826951

[B78] Wu W. Lv L. Shi D. Ye J. Fang D. Guo F.. (2017). Protective effect of akkermansia muciniphila against immune-mediated liver injury in a mouse model. Front. Microbiol. 8, 1804. 10.3389/fmicb.2017.01804 29033903 PMC5626943

[B79] Xiao N. Zhao Y. Yao Y. Wu N. Xu M. Du H.. (2020). Biological activities of egg yolk lipids: a review. J. Agric. Food Chem. 68, 1948–1957. 10.1021/acs.jafc.9b06616 32009394

[B80] Xue L. He J. Gao N. Lu X. Li M. Wu X.. (2017). Probiotics may delay the progression of nonalcoholic fatty liver disease by restoring the gut microbiota structure and improving intestinal endotoxemia. Sci. Rep. 7, 45176. 10.1038/srep45176 28349964 PMC5368635

[B81] Yanagita N. T. (2005). Dietary phosphatidylcholine alleviates fatty liver induced by orotic acid. Nutrition. 10.1016/j.nut.2004.11.019 15975496

[B82] Yang F. Ruan J. Wang T. Luo J. Cao H. Song Y.. (2017). Improving effect of dietary soybean phospholipids supplement on hepatic and serum indexes relevant to fatty liver hemorrhagic syndrome in laying hens. Anim. Sci. J. 88, 1860–1869. 10.1111/asj.12832 28677164

[B83] Yang M. Bose S. Lim S. Seo J. Shin J. Lee D.. (2020). Beneficial effects of newly isolated akkermansia muciniphila strains from the human gut on obesity and metabolic dysregulation. Microorganisms. 8, 1413. 10.3390/microorganisms8091413 32937828 PMC7564497

[B84] Yoo S. R. Kim Y. J. Park D. Y. Jung U. J. Jeon S. M. Ahn Y. T.. (2013). Probiotics L. plantarum and L. curvatus in combination alter hepatic lipid metabolism and suppress diet-induced obesity. Obesity (Silver Spring, Md). 21, 2571–2578. 10.1002/oby.20428 23512789

[B85] Yörük M. A. Gül M. Hayirli A. Laçin E. (2004). Laying performance and egg quality of hens supplemented with humate and sodium bicarbonate during the late laying period. J. Appl. Anim. Res. 26, 17–21. 10.1080/09712119.2004.9706498

[B86] Yu Z. Wang N. Ahn D. U. Ma M. (2019a). Long term egg yolk consumption alters lipid metabolism and attenuates hyperlipidemia in mice fed a high-fat diet based on lipidomics analysis. Eur. J. Lipid Sci. Technol. 121. 10.1002/ejlt.201800496

[B87] Yu Z. Wang S. Hou H. Ma L. Zhu Y. (2020). Lipidomic profiling reveals the effect of egg components on nonalcoholic steatosis in hepg2 cells and its involved mechanisms. Eur. J. Lipid Sci. Technol. 122. 10.1002/ejlt.201900451

[B88] Yu Z. Xiang X. Jin Y. Wang N. Ma M. (2019b). High-throughput lipidomic profiling of high-density lipoprotein from egg yolk (EYHDL): comparison based on UPLC-MS/MS and GC-MS. Eur. Food Res. Technol. 245, 1665–1675. 10.1007/s00217-019-03275-3

[B89] Zhang F. Liang Q. Xu X. Liu Z. Hui Z. Tao X.. (2017). Beneficial effects of probiotic cholesterol-lowering strain of Enterococcus faecium WEFA23 from infants on diet-induced metabolic syndrome in rats. J. Dairy Sci. 100, 1618–1628. 10.3168/jds.2016-11870 28041735

[B90] Zhang S. Zhao J. Xie F. He H. Johnston L. J. Dai X.. (2021). Dietary fiber-derived short-chain fatty acids: a potential therapeutic target to alleviate obesity-related nonalcoholic fatty liver disease. Obes. Rev. 22, e13316. 10.1111/obr.13316 34279051

[B91] Zhang T. Li Q. Cheng L. Buch H. Zhang F. (2019). Akkermansia muciniphila is a promising probiotic. Microb. Biotechnol. 12, 1109–1125. 10.1111/1751-7915.13410 31006995 PMC6801136

[B92] Zhang X. Zhao Y. Xu J. Xue Z. Zhang M. Pang X.. (2015). Modulation of gut microbiota by berberine and metformin during the treatment of high-fat diet-induced obesity in rats. Sci. Rep. 5, 14405. 10.1038/srep14405 26396057 PMC4585776

[B93] Zhang Z. F. Cho J. H. Kim I. H. (2013). Effects of Bacillus subtilis UBT-MO2 on growth performance, relative immune organ weight, gas concentration in excreta, and intestinal microbial shedding in broiler chickens. Livest. Sci. 155, 343–347. 10.1016/j.livsci.2013.05.021

[B94] Zhao R. Khafipour E. Sepehri S. Huang F. Beta T. Shen G. X. (2019). Impact of Saskatoon berry powder on insulin resistance and relationship with intestinal microbiota in high fat-high sucrose diet-induced obese mice. J. Nutr. Biochem. 69, 130–138. 10.1016/j.jnutbio.2019.03.023 31078906

[B95] Zhao S. Liu W. Wang J. Shi J. Sun Y. Wang W.. (2017). Akkermansia muciniphila improves metabolic profiles by reducing inflammation in chow diet-fed mice. J. Mol. Endocrinol. 58, 1–14. 10.1530/JME-16-0054 27821438

