# Supplementary material for: Akkermansia muciniphila Enhances Egg Quality and the Lipid Profile of Egg Yolk by Improving Lipid Metabolism
Source: Front Microbiol. 2022 Jul 19;13:927245. doi: 10.3389/fmicb.2022.927245 (PMC9344071; doi:10.3389/fmicb.2022.927245)
Supplement: Supplementary file 1 [file Data_Sheet_1.pdf]

Supplementary Material

Table S1 Effects of *A. muciniphila* treatment on egg quality of FLHS laying hens

Treatment	Egg weight /g	Egg shell strength/kgf	Albumen height/mm	Yolk color	Egg yolk ratio /%	Egg shell thickness /mm	Egg shape index
ND	65.14	4.18	8.39	6.67^{b}	26.46	0.77	1.26^{b}
HFD	63.81	4.35	7.73	7.73^{a}	28.19	0.78	1.30^{a}
HFD-LA	63.76	4.04	8.70	7.70^{a}	28.67	0.76	1.27^{b}
HFD-PA	63.54	4.21	8.91	7.93^{a}	27.11	0.79	1.28^{b}
SEM	0.28	0.07	0.19	0.08	0.39	0.004	0.003
P	0.181	0.417	0.150	<0.001	0.179	0.134	<0.001

^{a,b} Values with different superscripts within the same column differ significantly ($P < 0.05$), n=30.

Table S2 Identification of lipid biomarkers (PC and PE) between HFD and ND group

Num	Components	Log_2 (FC)	VIP	Model	Type
**HFD group versus ND group**					
1	PC 14:0_14:0	14.19	1.88	POS	up
2	PC 14:0_16:0	13.84	1.90	POS	up
3	PC 15:0_16:0	2.20	1.36	POS	up
4	PC 15:0_16:0 Esi+7.253536	14.60	1.83	POS	up
5	PC 15:0_22:5	3.00	1.10	POS	up
6	PC 15:0_22:5 Esi+7.1054277	13.41	1.53	POS	up
7	PC 16:0_16:0	13.63	1.75	POS	up
8	PC 16:0_16:3 Esi+5.068965	3.14	1.25	POS	up
9	PC 16:0_17:0	14.81	1.90	POS	up
10	PC 16:1_18:3 Esi+4.8476787	3.41	1.33	POS	up
11	PC 18:0_18:1	1.55	1.23	POS	up
12	PC 19:1_20:4 Esi+6.3958216	14.50	1.39	POS	up
13	PC 22:1_20:4 Esi+7.7075186	13.97	1.14	POS	up
14	PC 34:4 Esi+6.3364816	1.19	1.30	POS	up
15	PC 38:0	7.21	1.44	POS	up
16	PC 38:5	2.55	1.25	POS	up
17	PC 40:2	1.60	1.17	POS	up
18	PC 42:4	3.79	1.43	POS	up
19	PC 14:0_16:0	14.67	1.95	NEG	up
20	PC 14:0_20:4	2.46	1.24	NEG	up
21	PC 14:0_20:4 Esi-4.9812493	3.49	1.45	NEG	up
22	PC 14:0_22:5 Esi-6.8639793	14.73	1.52	NEG	up
23	PC 16:0_16:0	15.19	1.76	NEG	up
24	PC 17:0_18:2	5.66	1.45	NEG	up
25	PC 18:0_18:0	4.74	1.65	NEG	up
26	PC 18:1_20:4 Esi-6.5685825	14.86	1.11	NEG	up
27	PC 12:0_18:1	-3.35	1.63	POS	down
28	PC 15:0_18:2	-6.06	1.78	POS	down
29	PC 15:0_20:4 Esi+5.370216	-2.43	1.68	POS	down
30	PC 15:0_22:6	-2.90	1.75	POS	down
31	PC 16:0_16:1	-2.66	1.51	POS	down
32	PC 16:0_20:5	-4.49	1.89	POS	down
33	PC 16:1_18:2 Esi+5.3049464	-6.24	1.67	POS	down
34	PC 16:1_18:3	-3.70	1.93	POS	down
35	PC 16:1_20:4	-5.89	1.94	POS	down
36	PC 16:1_20:4 Esi+4.8929286	-1.98	1.21	POS	down
37	PC 16:1_20:4 Esi+5.404036	-3.54	1.78	POS	down
38	PC 16:3_20:4	-2.67	1.56	POS	down
39	PC 16:3_22:4 Esi+5.3603745	-3.94	1.73	POS	down
40	PC 18:0_20:3	-5.74	1.42	POS	down
41	PC 18:0_20:3 Esi+6.862695	-2.00	1.09	POS	down

42	PC 18:0_22:6 Esi+6.328303	-2.47	1.37	POS	down
43	PC 18:2_16:3	-5.23	1.92	POS	down
44	PC 18:2_20:4	-4.11	1.75	POS	down
45	PC 19:0_18:2	-4.21	1.77	POS	down
46	PC 19:0_20:4 Esi+6.9062314	-2.96	1.45	POS	down
47	PC 19:1_18:2	-7.80	1.35	POS	down
48	PC 20:1_22:6 Esi+6.369892	-4.53	1.34	POS	down
49	PC 22:1_20:4 Esi+7.3653035	-2.91	1.19	POS	down
50	PC 32:4	-3.88	1.80	POS	down
51	PC 33:3	-3.69	1.63	POS	down
52	PC 33:3 Esi+6.396428	-3.59	1.43	POS	down
53	PC 34:4	-3.86	1.71	POS	down
54	PC 35:7	-4.39	1.59	POS	down
55	PC 37:1	-4.82	1.83	POS	down
56	PC 38:9	-3.74	1.86	POS	down
57	PC 41:6	-3.36	1.73	POS	down
58	PC 42:8	-4.92	1.64	POS	down
59	PC 42:8 Esi+6.0643935	-2.21	1.37	POS	down
60	PC 14:0_18:2	-2.30	1.11	NEG	down
61	PC 14:0_20:4 Esi-4.818271	-3.15	1.52	NEG	down
62	PC 14:0_20:4 Esi-5.323729	-3.41	1.55	NEG	down
63	PC 14:0_22:5	-3.41	1.80	NEG	down
64	PC 14:0_22:5 Esi-4.8958535	-7.19	1.91	NEG	down
65	PC 14:0_22:5 Esi-5.5695424	-3.78	1.82	NEG	down
66	PC 15:0_18:2 Esi-5.4222713	-5.80	1.78	NEG	down
67	PC 16:0_16:1	-3.97	1.82	NEG	down
68	PC 16:0_20:4 Esi-5.388458	-9.92	1.74	NEG	down
69	PC 16:1_18:2	-3.71	1.27	NEG	down
70	PC 16:1_18:2 Esi-5.429521	-4.95	1.40	NEG	down
71	PC 16:1_18:2 Esi-5.429521 :1	-4.95	1.40	NEG	down
72	PC 16:1_22:6	-2.12	1.46	NEG	down
73	PC 16:1_22:6 Esi-5.5231113	-3.35	1.14	NEG	down
74	PC 17:1_18:2	-1.64	1.14	NEG	down
75	PC 17:1_18:2 Esi-7.298021	-1.76	1.23	NEG	down
76	PC 18:0_18:1	-4.81	1.71	NEG	down
77	PC 18:0_22:4 Esi-8.161937	-2.30	1.20	NEG	down
78	PC 18:1_22:5	-3.38	1.56	NEG	down
79	PC 18:1_22:5 Esi-6.919166	-2.39	1.46	NEG	down
80	PC 18:2_20:4	-2.71	1.63	NEG	down
81	PC 19:0_18:2	-3.98	1.65	NEG	down
82	PE 17:0_18:1 Esi+6.9808035	1.95	1.42	POS	up
83	PE 18:0_18:0	14.32	1.76	POS	up
84	PE 18:0_18:0 Esi+8.098143	3.36	1.42	POS	up
85	PE 18:0_20:1	13.59	1.69	POS	up
86	PE 18:0_22:4	13.43	1.68	POS	up
87	PE 32:0	1.48	1.19	POS	up

88	PE 40:3 Esi+7.950071	1.91	1.22	POS	up
89	PE 16:0_16:0	2.93	1.16	NEG	up
90	PE 16:0_16:0 Esi-3.8361936	14.73	2.03	NEG	up
91	PE 16:0_18:0	15.00	1.83	NEG	up
92	PE 16:0_18:1 Esi-3.9060564	15.39	2.03	NEG	up
93	PE 16:0_18:2 Esi-3.5353327	2.71	1.33	NEG	up
94	PE 18:0_18:0	1.49	1.16	NEG	up
95	PE 18:0_20:1	3.60	1.39	NEG	up
96	PE 18:0_20:1 Esi-8.340333	3.90	1.37	NEG	up
97	PE 18:0_22:5	5.00	1.32	NEG	up
98	PE 16:0_16:1	-2.48	1.56	POS	down
99	PE 16:0_20:4	-15.46	1.64	POS	down
100	PE 16:1_22:6	-4.85	1.77	POS	down
101	PE 18:0_20:3 Esi+7.17916	-1.67	1.27	POS	down
102	PE 18:1_16:3	-3.61	1.65	POS	down
103	PE 18:1_18:2	-4.04	1.21	POS	down
104	PE 18:2_16:3	-5.37	1.92	POS	down
105	PE 36:5	-5.84	1.84	POS	down
106	PE 36:5 Esi+5.218839	-6.87	1.74	POS	down
107	PE 16:0_16:1	-3.67	1.76	NEG	down
108	PE 16:0_17:1	-4.59	1.82	NEG	down
109	PE 16:0_18:2	-2.84	1.07	NEG	down
110	PE 16:0_22:4 Esi-5.3640003	-4.02	1.55	NEG	down
111	PE 16:0_22:4 Esi-7.6325417	-2.84	1.17	NEG	down
112	PE 16:0_22:4 Esi-7.6325417 :1	-2.84	1.17	NEG	down
113	PE 16:0_22:6	-2.52	1.36	NEG	down
114	PE 16:1_18:2 Esi-4.9229293	-2.87	1.72	NEG	down
115	PE 18:0_24:4 Esi-7.077334	-2.36	1.19	NEG	down
116	PE 18:1_16:3	-3.25	1.46	NEG	down
117	PE 18:1_18:2 Esi-6.3896174	-3.52	1.40	NEG	down
118	PE 18:2_16:3	-5.22	1.94	NEG	down
119	PE 18:2_18:2 Esi-5.64275	-7.51	1.63	NEG	down
120	PE 18:2_18:3	-5.30	1.87	NEG	down
121	PE 18:2_18:3 Esi-5.127479	-8.02	1.80	NEG	down
122	PE 18:2_18:3 Esi-5.546354	-3.08	1.31	NEG	down
123	PE 18:2_18:3 Esi-5.8334794	-4.63	1.78	NEG	down
124	PE 18:3_20:4	-3.47	1.71	NEG	down
125	PE 18:3_20:4 Esi-4.752458	-3.07	1.59	NEG	down
126	PE 18:3_20:4 Esi-5.7930818	-4.09	1.76	NEG	down
127	PE 19:0_18:2	-5.21	1.55	NEG	down
128	PE 19:0_18:2 Esi-7.4061666	-4.27	1.18	NEG	down

The change trend of potential biomarkers labeled with (up) and (down) correspondingly stands for increase and decrease, respectively ($P < 0.05$). Data were analyzed by t-test, n=10.

Table S3 Identification of lipid biomarkers (TG and PE) between HFD and HFD_LA group

Num	Components	Log_2 (FC)	VIP	Model	Type
**HFD group versus HFD_LA group**					
1	TG 16:0_16:0_16:0	4.52	1.59	POS	up
2	TG 16:0_16:0_18:0	3.41	1.65	POS	up
3	TG 16:0_16:0_18:1	3.14	1.63	POS	up
4	TG 16:0_16:1_18:1 Esi+13.018662	11.97	1.61	POS	up
5	TG 18:0_18:0_18:0	2.53	1.53	POS	up
6	TG 18:0_18:0_18:0 Esi+13.79865	14.39	1.74	POS	up
7	TG 48:3	1.93	1.52	POS	up
8	TG 52:1 Esi+13.83777	3.22	1.78	POS	up
9	TG 55:2 Esi+13.270089	2.45	1.52	POS	up
10	TG 56:1 Esi+14.497357	1.48	1.80	POS	up
11	PE 16:0_20:4 Esi+5.8885713	1.90	1.71	POS	up
12	PE 16:0_16:0 Esi-3.8361936	1.35	1.05	NEG	up
13	PE 16:0_22:6 Esi-5.5941453	2.23	2.34	NEG	up
14	PE 16:1_18:2 Esi-4.9229293	1.26	2.04	NEG	up
15	PE 18:0_19:1	2.67	1.96	NEG	up
16	PE 18:1_16:3 Esi-5.2230864	1.17	2.02	NEG	up
17	PE 16:0_22:6 Esi-5.144422	-2.38	1.82	NEG	down
18	PE 18:0_19:1 Esi-7.548595	-1.87	1.46	NEG	down
19	PE 18:0_19:1 Esi-7.983374	-2.56	1.35	NEG	down
20	PE 18:0_22:3	-2.54	1.41	NEG	down
21	PE 18:0_22:3 Esi-7.952938	-3.44	1.79	NEG	down
22	PE 18:0_22:5	-1.31	1.18	NEG	down
23	PE 18:0_24:4	-2.78	1.92	NEG	down
24	PE 18:1_20:4	-1.12	1.43	NEG	down

The change trend of potential biomarkers labeled with (up) and (down) correspondingly stands for increase and decrease, respectively ($P < 0.05$). Data were analyzed by t-test, n=10.

Table S4 Identification of lipid biomarkers (TG and DG) between HFD and HFD_PA group

Num	Components	Log_2 (FC)	VIP	Model	Type
**HFD group versus HFD_PA group**					
1	TG 16:0_18:2_18:2 Esi+12.726323	1.80	1.45	POS	up
2	TG 16:0_18:2_18:2 Esi+13.384588	3.30	1.54	POS	up
3	TG 17:1_18:2_18:3	1.82	1.45	POS	up
4	TG 18:0_18:1_19:0	3.10	1.51	POS	up
5	TG 46:1	2.48	1.67	POS	up
6	TG 48:1	2.31	1.68	POS	up
7	TG 49:2	1.63	1.53	POS	up
8	TG 51:2 Esi+13.271359	2.80	1.61	POS	up
9	TG 52:1	1.12	1.53	POS	up
10	TG 52:1 Esi+13.799945	2.08	1.65	POS	up
11	TG 53:2 Esi+13.71316	1.63	1.70	POS	up
12	TG 53:3 Esi+13.334497	2.35	1.53	POS	up
13	TG 54:1 Esi+13.388322	3.36	1.38	POS	up
14	TG 56:2 Esi+13.266231	1.51	1.49	POS	up
15	TG 56:7	3.59	1.60	POS	up
16	TG 12:0_12:0_12:0 Esi+9.8955	-1.80	1.36	POS	down
17	TG 14:1_18:1_18:2	-3.03	1.40	POS	down
18	TG 15:0_16:1_18:2	-3.07	1.27	POS	down
19	TG 15:0_16:1_18:3	-3.95	2.06	POS	down
20	TG 15:0_16:1_18:3 Esi+12.0668	-1.84	1.36	POS	down
21	TG 16:0_16:1_22:6	-2.52	1.35	POS	down
22	TG 16:0_17:1_18:2 Esi+12.103248	-3.51	1.50	POS	down
23	TG 16:0_17:1_18:2 Esi+12.88191	-1.82	1.56	POS	down
24	TG 16:0_17:1_20:4	-1.94	1.49	POS	down
25	TG 16:0_18:1_20:4	-2.17	1.69	POS	down
26	TG 16:0_18:1_22:6	-1.96	1.51	POS	down
27	TG 16:0_18:2_18:2	-3.72	1.75	POS	down
28	TG 16:0_18:2_18:2 Esi+12.737438	-2.67	1.70	POS	down
29	TG 16:0_18:2_18:2 Esi+12.85	-1.78	1.36	POS	down
30	TG 16:0_18:2_22:6	-2.18	1.51	POS	down
31	TG 16:0_20:4_22:6	-1.93	1.49	POS	down
32	TG 16:1_17:1_18:2	-2.19	1.61	POS	down
33	TG 17:0_18:0_20:4	-3.06	1.37	POS	down
34	TG 17:0_18:1_20:4	-2.21	1.84	POS	down
35	TG 17:0_18:1_20:4 Esi+13.273806	-1.22	1.45	POS	down
36	TG 17:1_18:1_18:2 Esi+12.931569	-1.92	1.70	POS	down
37	TG 18:0_18:0_22:4 Esi+14.064618	-5.17	1.96	POS	down
38	TG 18:0_18:1_18:1 Esi+13.920318	-1.74	1.50	POS	down
39	TG 18:0_18:1_20:1	-1.89	1.54	POS	down
40	TG 18:0_18:1_22:5	-2.41	2.00	POS	down
41	TG 18:0_18:1_22:5 Esi+13.371247	-3.90	1.86	POS	down

42	TG 18:4_22:0_22:2 Esi+14.153788	-6.21	1.97	POS	down
43	TG 46:3 Esi+11.527314	-2.66	1.45	POS	down
44	TG 46:3 Esi+11.625999	-1.84	1.36	POS	down
45	TG 50:5	-2.50	1.35	POS	down
46	TG 52:6 Esi+12.22827	-1.52	1.41	POS	down
47	TG 53:1 Esi+14.052894	-1.31	1.29	POS	down
48	TG 53:3 Esi+13.339037	-1.54	1.56	POS	down
49	TG 54:1 Esi+14.243482	-1.34	1.30	POS	down
50	TG 54:7	-2.36	1.44	POS	down
51	TG 55:2 Esi+14.084055	-1.88	1.51	POS	down
52	TG 56:3	-2.17	1.64	POS	down
53	TG 56:5	-12.66	1.69	POS	down
54	TG 56:5 Esi+13.360824	-2.44	1.67	POS	down
55	DG 18:1_24:4	1.35	1.51	POS	up
56	DG 16:1_18:1 Esi+7.748	-1.80	1.36	POS	down
57	DG 16:1_18:1 Esi+7.914963	-5.73	1.87	POS	down
58	DG 16:1_18:1 Esi+7.9758573	-8.85	1.45	POS	down
59	DG 17:1_18:1 Esi+8.421	-1.80	1.36	POS	down
60	DG 18:1_18:1 Esi+8.629	-1.80	1.36	POS	down
61	DG 18:1_18:2	-2.77	1.46	POS	down
62	DG 18:1_22:6	-1.67	1.29	POS	down
63	DG 18:1_24:4 Esi+9.259554	-2.70	1.61	POS	down
64	DG 18:1_24:4 Esi+9.337447	-2.42	1.33	POS	down
65	DG 18:2_18:2 Esi+7.467606	-4.40	1.70	POS	down
66	DG 18:2_18:2 Esi+7.5193396	-3.66	1.31	POS	down

The change trend of potential biomarkers labeled with (up) and (down) correspondingly stands for increase and decrease, respectively ($P < 0.05$). Data were analyzed by t-test, n=10.

Table S5 Effects of *A. muciniphila* addition on production performance of aged laying hens

Items	Treatment		*P*-value
	Control	*A. muciniphila*	
Laying rate / %			
Weeks 65-68	90.46±0.99	90.00±0.80	0.652
Weeks 69-72	89.13±1.84	88.98±0.65	0.917
Weeks 73-76	85.26±1.87	86.33±1.14	0.426
Average egg weight / g			
Weeks 65-68	62.76±0.51	62.82±0.46	0.825
Weeks 69-72	63.35±0.48	63.63±0.54	0.394
Weeks 73-76	63.70±0.37	63.88±0.37	0.629
Average daily feed intake/ g			
Weeks 65-68	115.18±5.66	113.87±6.53	0.773
Weeks 69-72	111.71±9.25	110.94±10.36	0.915
Weeks 73-76	104.66±17.20	103.97±15.56	0.955
Feed to gain ratio			
Weeks 65-68	2.04±0.08	2.02±0.10	0.841
Weeks 69-72	1.99±0.20	1.98±0.18	0.941
Weeks 73-76	1.94±0.33	1.91±0.28	0.888

Values are the mean ± standard deviation, n=7.

Table S6 Effects of *A. muciniphila* addition on egg quality of aged laying hens

Items	Egg weight /g	Egg shell strength /kgf	Albumen height /mm	Yolk color
Control	62.02±3.61	3.42±0.90	6.61±1.80	6.88±1.15
A. muciniphila	63.22±3.56	3.39±0.94	6.98±1.16	7.06±1.15
P-value	0.051	0.808	0.139	0.362

Values are the mean ± standard deviation, n=7.

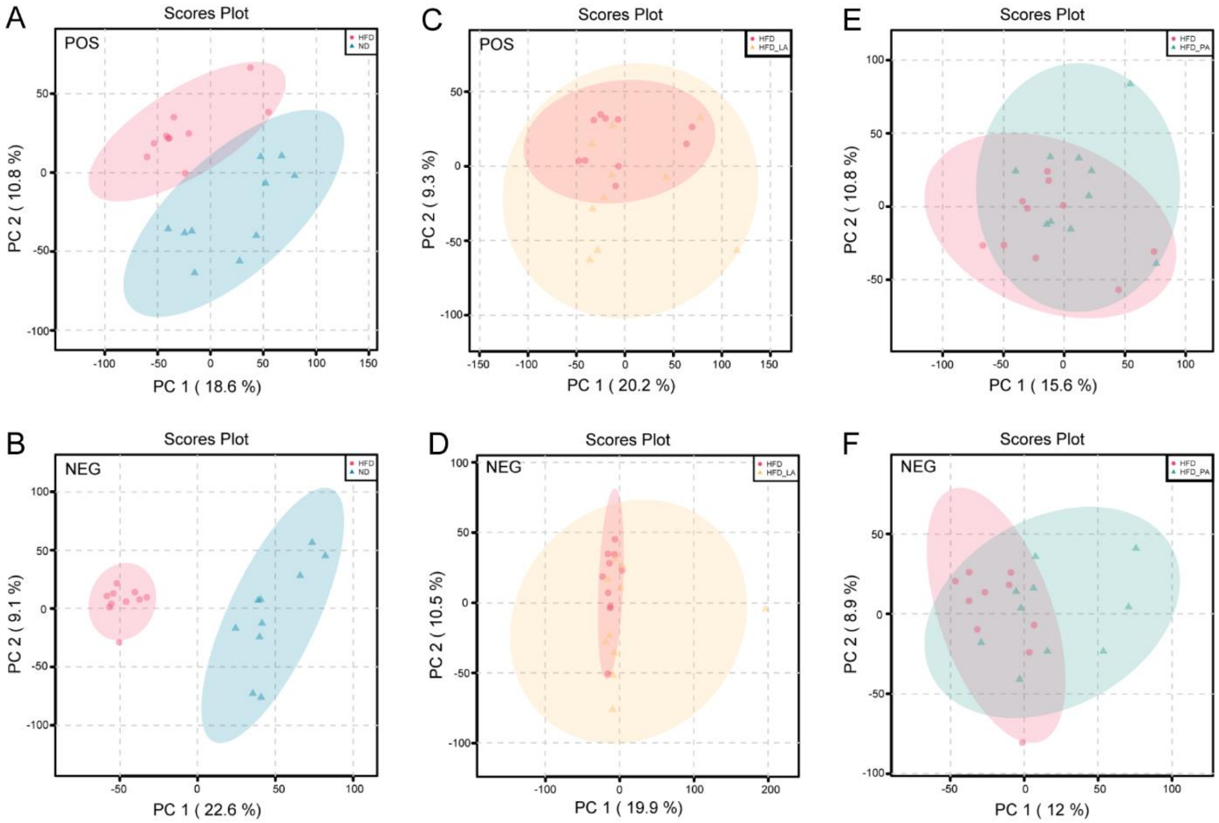

Figure S1. Principal component analysis (PCA) score plots of egg yolk samples (n=10). (A and B) The PCA score plots between ND and HFD group in ES+ and ES-modes. (C and D) The PCA score plots between HFD and HFD_LA group in ES+ and ES-modes. (E and F) The PCA score plots between HFD and HFD_PA group in ES+ and ES-modes. POS represents ES+ modes, and NEG represents ES- modes.

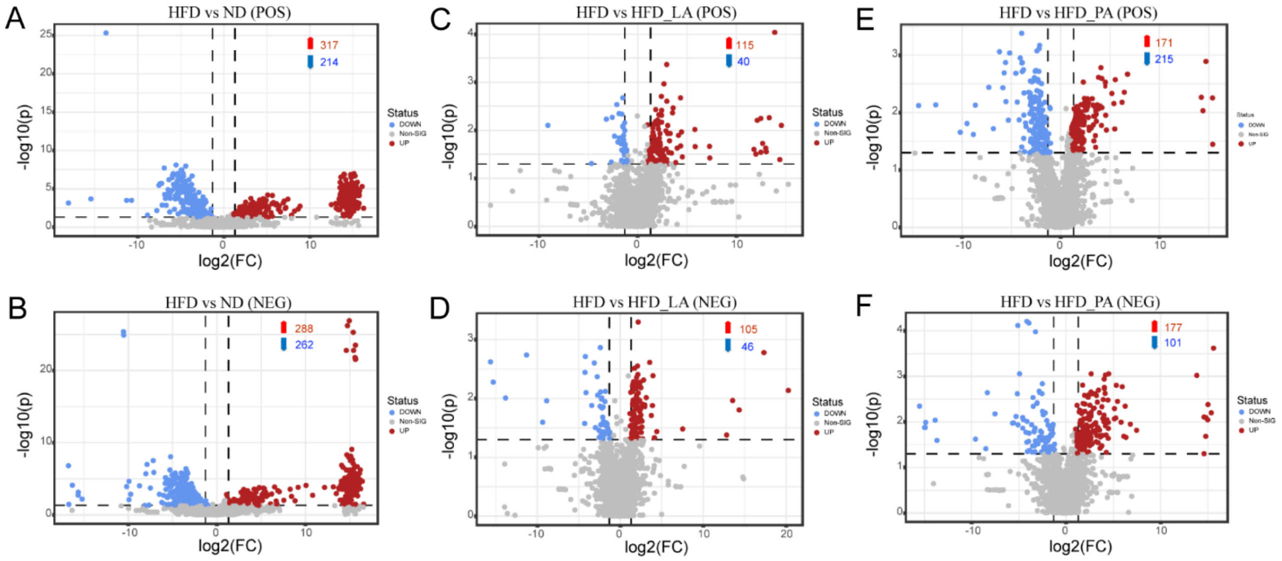

Figure S2. The volcano plots of egg yolk samples (n=10). (A and B) The volcano plots between ND and HFD group in ES+ and ES-modes. (C and D) The volcano plots between HFD and HFD_LA group in ES+ and ES-modes. (E and F) The volcano plots between HFD and HFD_PA group in ES+ and ES-modes. POS represents ES+ modes, and NEG represents ES- modes. Red represents upregulated lipid species, and blue represents downregulated lipid species, including both annotated and not annotated.
